# ABO blood group antigens influence host–microbe interactions and risk of early spontaneous preterm birth

**DOI:** 10.1038/s41522-025-00783-x

**Published:** 2025-09-10

**Authors:** Katherine E. Mountain, David A. MacIntyre, Yun S. Lee, Virginia Tajadura-Ortega, Anne Dell, Stuart M. Haslam, Gang Wu, Paola Grassi, Ten Feizi, Yan Liu, Wengang Chai, Julian R. Marchesi, Lauren A. Roberts, Denise Chan, Belen Gimeno-Molina, Richard G. Brown, Holly V. Lewis, Alice K. C. Hyde, James Pasint-Magyar, Anna Green, Anna L. David, Jane E. Norman, Sarah J. Stock, Samit Kundu, Sherrianne Ng, Ben Glampson, Erik Mayer, TG Teoh, Vasso Terzidou, Phillip R. Bennett, Lynne Sykes

**Affiliations:** 1 Imperial College Parturition Research Group, Institute of Reproductive and Developmental Biology, Department of Metabolism, Digestion and Reproduction, Imperial College London, London, UK; 2 March of Dimes Prematurity Research Center at Imperial College London, London, UK; 3 Parasol Foundation Centre for Women’s Health and Cancer Research, St Mary’s Hospital, Imperial College Healthcare NHS Trust, London, UK; 4 Robinson Research Institute, University of Adelaide, Adelaide, Australia; 5 Glycosciences Laboratory, Department of Metabolism, Digestion and Reproduction, Imperial College London, London, UK; 6 Department of Life Sciences, Imperial College London, London, UK; 7 Division of Digestive Diseases, Department of Metabolism, Digestion and Reproduction, Imperial College London, London, UK; 8 Elizabeth Garrett Anderson Institute for Women’s Health, University College London, London, UK; 9 University of Nottingham, Nottingham, UK; 10 University of Edinburgh, Edinburgh, UK; 11 Digital Collaboration Space, Imperial Clinical Analytics, Research & Evaluation (iCARE) Secure Data Environment, Imperial College London & Imperial College Healthcare NHS Trust, London, UK; 12 Chelsea & Westminster Hospital, Imperial College Healthcare NHS Trust, London, UK

**Keywords:** Bacteria, Clinical microbiology, Microbiology, Health care

## Abstract

The mechanisms by which vaginal microbiota shape spontaneous preterm birth (sPTB) risk remain poorly defined. Using electronic clinical records data from 74,913 maternities in conjunction with metaxanomic (*n* = 596) and immune profiling (*n* = 314) data, we show that the B blood group phenotype associates with increased risk of sPTB and adverse vaginal microbiota composition. The O blood group associates with sPTB in women who have a combination of a previous history of sPTB, an adverse vaginal microbial composition and pro-inflammatory cervicovaginal *milieu*. In contrast, women of blood group A have a higher prevalence of vaginal *Lactobacillus crispatus*, a lower risk of sPTB, with sPTB cases showing no association with vaginal microbiota composition or inflammation. We found that cervicovaginal fluid contains ABH(O) glycans and shows variable binding to key vaginal bacteria. This indicates that cervicovaginal ABH(O) glycans influence microbiota-host interactions implicated in sPTB risk, suggesting a novel target for sPTB prediction and prevention.

## Introduction

Preterm birth (PTB) occurs in 10% of pregnancies, affecting 13.4 million babies. It is the leading cause of mortality in newborns and children under the age of five^1,2^. Spontaneous preterm birth (sPTB), is preceded by the onset of premature contractions, cervical shortening and foetal membrane rupture, and accounts for over half of PTBs^3^, with the rest classified as iatrogenic (iPTB) being medically indicated for either maternal or foetal reasons. The causes of PTB are multifactorial, with known risk factors including previous premature birth and previous cervical excisional treatment (LLETZ) for pre-cancerous cells. Cervical mechanical weakness is a contributing factor in women who have had LLETZ. In other cases, the most common causal mechanisms are linked to adverse vaginal microbiota and/or activation of inflammation. This is particularly the case for early sPTB <34 weeks, where highest rates of neonatal morbidity and mortality are seen^3–6^. Despite this, common current treatments to prevent sPTB, progesterone and cervical cerclage, have not been shown to improve the vaginal microbiome or have a beneficial effect upon the immune *milieu*^7–9^.

During pregnancy, high oestrogen levels facilitate a shift towards a stable vaginal microbial composition dominated by *Lactobacillus* spp^10^. This creates an acidic environment, which limits polymicrobial diversity. A vaginal microbial composition dominated by *Lactobacillus crispatu*s, often referred to as Community State Type I (CST I)^11^, is protective against sPTB^7,12–14^, whereas women with *L. iners* dominance (CST III), or a depletion of *Lactobacillus* species and polymicrobial composition (CST IV) are at a higher risk^7,8,15^. The cervicovaginal immune *milieu* is a key factor driving the process of microbial-associated sPTB. CST III and IV both associate with higher concentrations of cervicovaginal fluid (CVF) cytokine and complement proteins when compared to CST I. Further increases are seen in women who deliver preterm compared to term^8,9,15^. Despite a clear association between vaginal microbial composition and the host immune response, the factors influencing bacterial composition and individual variations in immune responses and pregnancy outcome are poorly understood.

The histo-blood groups (ABO) are the most widely known glycan structures, with four main types—A, B, AB, and O^16^. The A, B, and O (H) blood group antigens are presented on Type-1 and Type-2 antennae of O- or N-glycans and Type-3 backbones displayed on O-glycans. The presence of ABO blood group phenotypes is reliant on the synthesis of ABO(H) antigens under the regulation of two genes: *FUT1* and *ABO*. The precursor of the A and B antigens is the H antigen, the production of which is controlled by the *FUT1* gene^17^. *FUT1* encodes a specific fucosyltransferase (α1-2FucT) which adds fucose (Fuc) in an α1-2 linkage to the terminal galactose of the precursor. The ABO genes encode glycosyltransferases enzyme that determine the modification of the H antigen and ultimately the production of A and B antigen. The α1-3GalNAcT (or Enzyme A) generates the A epitope by adding a N-acetylgalactosamine (GalNAc) residue to the H antigen; α1-3GalT (or Enzyme B) forms the B blood group by adding a galactose (Gal) residue to the H antigen. Unmodified H antigens correspond to the O blood group phenotype. As well as being produced on red blood cells, ABO(H) antigens are also synthesised by epithelial cells, including those within the vagina and cervix^18,19^. This starts with the action of the glycosyltransferases (*FUT2)*, which adds an α1-2-linked Fuc to Gal to form the blood group H antigen on Type-1 and Type-2 chains on N- and O-glycans. The glycan structures of the ABH antigens are shown in Supplementary Fig. 1.

ABO(H) antigens are secreted into the bodily fluids of individuals at varying amounts depending on blood group and secretor status^20,21^. ABO blood group phenotype associates with susceptibility to pathogen-driven disease, since the ABO(H) antigens can act as adhesion molecules and a source of nutrients for bacteria, whereas the presence of anti-A, B and H antibodies can offer protection or immunogenicity^22^. For example, blood group O individuals are more susceptible to infection by *Helicobacter pylori*, whereas blood group B individuals are more susceptible to *Neisseria gonorrhoea*, *Escherichia coli*, and Group B *Streptococcus* infection^22–24^, the latter known to be associated with an increased risk of sPTB^25,26^. The ABO blood group phenotype has also shown differential associations with the gut microbiome structure^27–29^, with preferential binding of certain *Lactobacilli* strains to the A antigen and gut epithelial surfaces of blood group A individuals^28,29^.

We hypothesised that blood group A individuals are more likely to have *L. crispatus* colonisation, conferring protection from sPTB, whereas blood group O and B individuals are at increased risk of vaginal microbial diversity and thus early sPTB. This study reveals an association between ABO blood group phenotype, microbial composition, immune *milieu* and sPTB risk. These findings provide mechanistic insights with the potential to lead to glycan-directed therapeutics, live biotherapeutics and immune modulators in a targeted individualised approach for preterm birth prevention.

## Results

### Study population

**Fig. 1:**
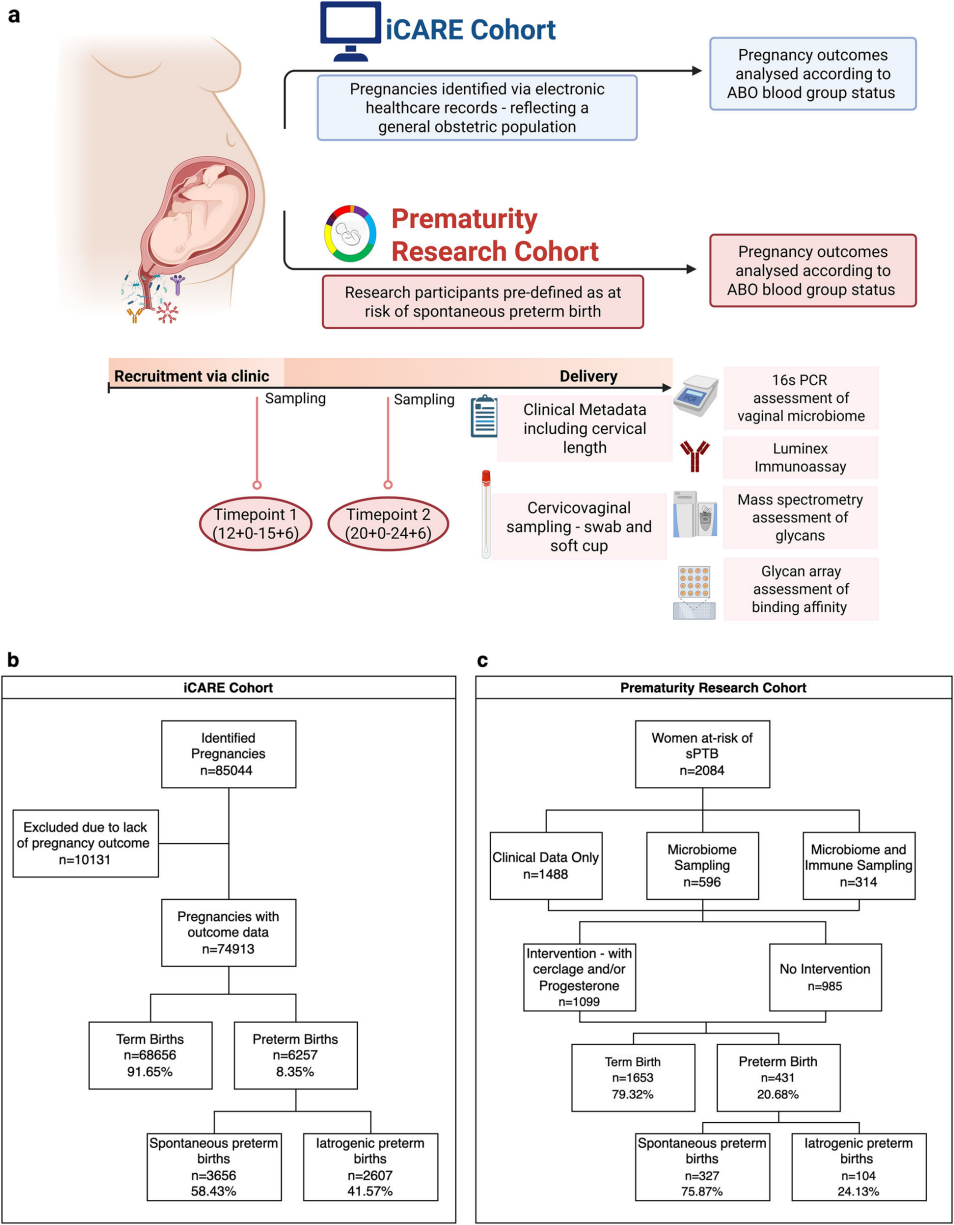
Summary of study design and study population. **a** Schematic representing study design: The iCARE study population included maternities identified via electronic healthcare records representing general population and the Prematurity Research study population included maternities with a pre-defined risk factor for spontaneous preterm birth (sPTB), a proportion of whom provided samples for vaginal microbial composition, cervicovaginal (CVF) immune mediators and CVF glycan profiles. **b** Pregnancy outcomes and ABO blood group status were analysed from a general obstetric population (iCARE population) across three inner London Hospitals between April 2014 and x 2023 (85,044 maternities). **c** Pregnancy outcomes and ABO blood group status were analysed from the Prematurity Research cohort of women defined as at risk of sPTB and/or who participated in the multi-centre VMET-2 study at four inner London hospitals, and Edinburgh (*n* = 2084).

A total of 85,044 maternities from an inner London population were identified from electronic patient records (iCARE Secure Data Environment) (Fig. 1a). The PTB rate was 8.35%, 58.43% were spontaneous (sPTB) and 41.57% were iatrogenic (iPTB) (Fig. 1b). A second population of 2084 women at increased risk of sPTB were prospectively recruited from Preterm Birth Prevention Clinics (Fig. 1a) and had a PTB rate of 20.68%, 75.87% were spontaneous and 24.13% were iatrogenic, reflecting a population enriched with women at high-risk of sPTB (Fig. 1c). Demographic data according to ABO blood type is available in Supplementary Tables 1 and 2.

### ABO blood group phenotype and risk of PTB

**Fig. 2:**
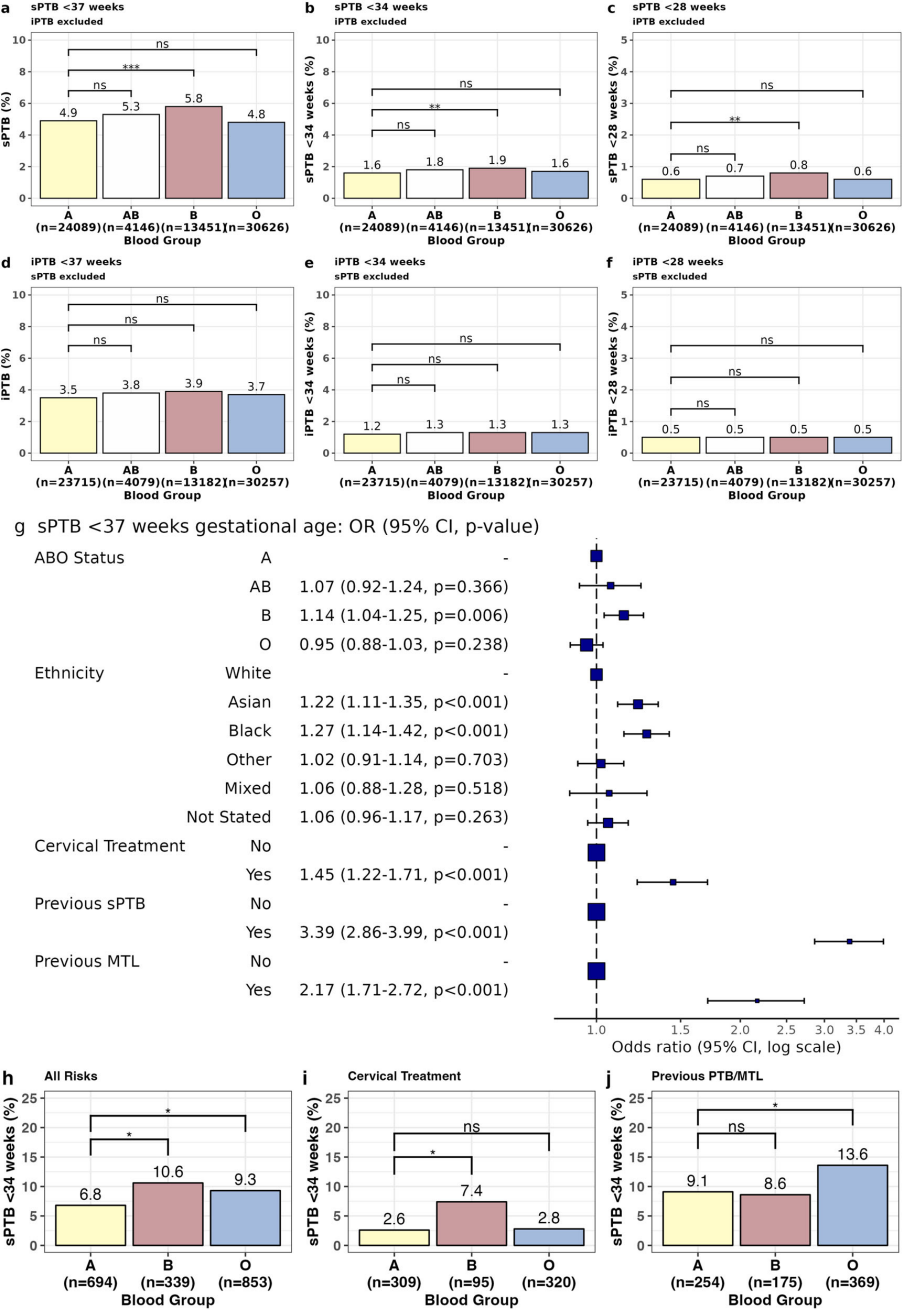
Pregnancy outcomes according to ABO blood group. In the iCARE cohort of 74,913 maternities with pregnancy outcomes, the rates of spontaneous preterm birth by ABO group are shown for (**a**) sPTB < 37 weeks (**b**) <34 weeks and (**c**) <28 weeks. No relationship between ABO blood group and iatrogenic preterm birth is seen (**d**) <37 weeks (**e**) < 34 weeks and (**f**) <28 weeks. **g** Adjusted odds model for sPTB < 37 weeks generated from logistic regression modelling (*n* = 74,913). Outcome was adjusted for blood group, ethnicity, past pregnancy history and history of previous cervical treatment. There was no improvement to the model with the addition of interaction terms between co-variates. **h** In women with a pre-defined risk factor, sPTB rates are increased in women with blood group B and O phenotype (*n* = 1886. all-risk factors), (**i)** and in blood group B with previous cervical treatment *n* = 724), **j** but in women with a history of previous sPTB and MTL an increased risk is seen in women of blood group O phenotype (*n* = 798). N.B. Previous PTB/MTL population excludes those with concurrent uterine anomaly or previous cervical treatment. Comparisons made using Chi-squared test and adjusted for multiple comparisons. *P* value significance: *<0.05, **<0.01, ***<0.001, ****<0.0001. Adjusted OR ± 95% CI and *p* value.

There was a significant increase in the rate of sPTBs in women of blood group B phenotype compared with blood group A, 5.8% vs 4.9% (*p* = 0.001); 1.9% vs 1.6% (*p* = 0.009); 0.8% vs 0.6% *(p* = 0.006) at the thresholds of gestational age <37, <34 and <28 weeks, respectively, in the general maternity population (Fig. 2a–c). An increase in the need for cervical cerclage intervention was observed in blood group B women compared with blood group A women, 1.4% vs 0.9% (*p* < 0.001) (Supplementary Table 3). There were no differences in iatrogenic PTB rates across ABO blood groups (Fig. 2d–f).

ABO blood group phenotype and established risk factors for sPTB were tested in an adjusted odds model for sPTB < 37 weeks using logistic regression modelling (Fig. 2g). The adjusted odds ratios (OR) were as follows; B blood group phenotype 1.14 (*p* = 0.006), Asian and Black ethnicity 1.22 (*p* < 0.001) and 1.27 (*p* < 0.001) respectively, a history of previous cervical treatment 1.45 (*p* < 0.001), sPTB 3.39 (*p* < 0.001), and mid-trimester loss (MTL) 2.17 (*p* < 0.001). Whilst there were significant differences between the distribution of blood groups and ethnicities, an interaction between these co-variates did not significantly affect the overall model. In the high-risk population, compared to blood group A, women of blood group B had an increase in the risk of sPTB <34 weeks 10.6% vs 6.8% (*p* = 0.016) (Fig. 2h), sPTB <28 weeks (*p* = 0.01) and cervical shortening (*p* = 0.02) (Supplementary Table 4), OR 1.63, 2.01 and 1.35 respectively. In addition, there was a significant increase in the rates of sPTB and MTL in women of blood group O compared to blood group A, 9.3% vs 6.8% (*p* = 0.037), OR 1.40 and 1.17, respectively (Fig. 2h, Supplementary Table 4).

In women with a history of cervical treatment, higher rates of sPTB (7.4% vs 2.6%, *p* = 0.02) (Fig. 2i) and cervical shortening (22.1% vs 13.6%, *p* = 0.02) were seen in blood group B compared to blood group A, however this was not seen in women with a history of sPTB. In women with a history of sPTB, an increase in sPTB rate was seen only in women with blood group O phenotype (13.6% blood group O vs 9.1% in blood group A) (*p* = 0.04) (Fig. 2j). Collectively this implies that the different ABO blood group phenotypes may contribute to sPTB aetiology by different mechanisms and suggests that blood group A appears to be protective of cervical shortening, the need for intervention, and PTB.

### ABO(H) glycans at the cervicovaginal interface

**Fig. 3:**
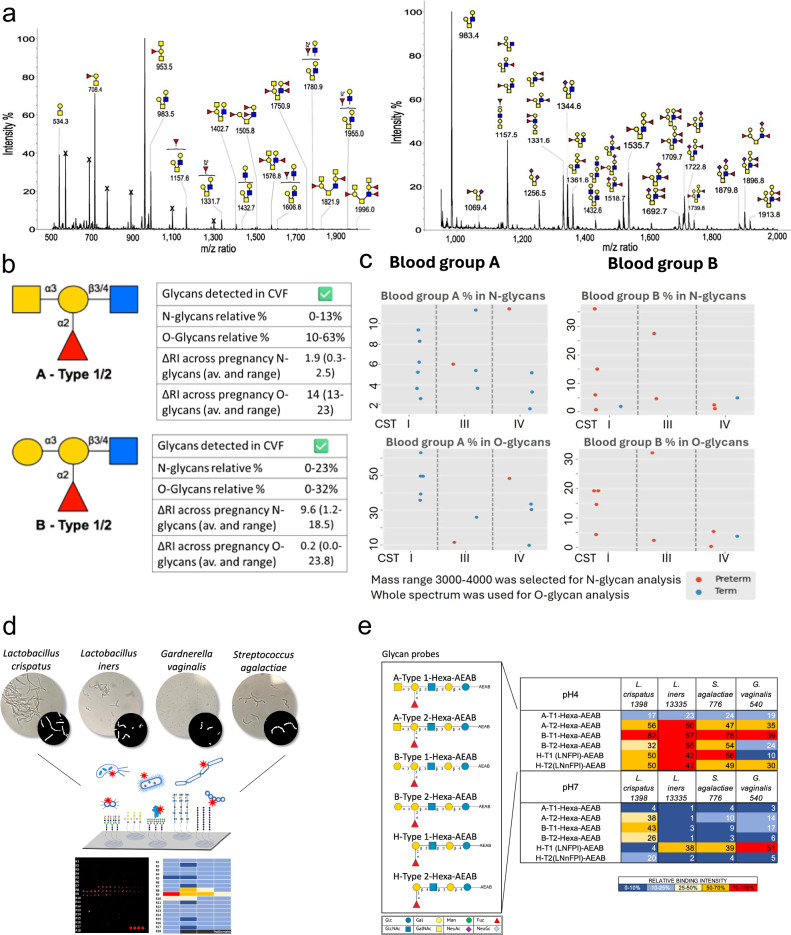
ABO blood group glycans at the cervicovaginal interface and interaction with common vaginal microbiota. **a** Representative MALDI-TOF mass spectrum of O-glycans released from CVF of an A blood group and B blood group donors. Assignments are based on composition, tandem MS and knowledge of biosynthetic pathways. All molecular ions are [M+Na]^+^. **b** Semi-quantitative analyses of whole spectrum show higher percentages of A and B blood group epitopes expressed on O-glycans than on N-glycans. *n* = 64. **c** The highest abundance of the A blood group antigen on glycans isolated from CVF is detected in association with CST-I and term delivery, whereas the presence of the B antigen, which is lower in abundance, is seen across all CSTs, and is predominantly seen in women who have a preterm delivery. Mass range 3000-4000 for N-glycan analysis, whole spectrum for O-glycan analysis. *n* = 46. **d** A schema of glycan microarrays constructed to detect glycan binding with fluorescently labelled in-house cultured bacterial isolates of species and strains. **e** Heatmap showing the relative binding intensities to blood group glycan probes at pH4 and pH7 of fluorescently labelled bacteria. Differential binding of commensals and pathobionts is seen depending on ABH backbone and pH. The average rank of mean fluorescence intensities of quadruplicate spots from three binding assays was calculated and the resulting values coloured as follows: Dark blue (0-10%); Light blue (10-25%); Yellow (25-50%); Orange (50-70%); Red (70-100%). 100%, the maximum binding score observed across pH4 and pH7 for a given strain.

MALDI-TOF MS based glycomic analyses^30,31^ of CVF from pregnant women confirmed the presence of the H antigen (Fuc-Gal-) in O blood group individuals, the A antigen (GalNAc-(Fuc-)Gal) in blood group A individuals and the B antigen (Gal-(Fuc-)Gal) in blood group B individuals (Fig. 3a). ABO(H) epitopes were mainly expressed on O-glycans and were detected at varying levels on N and O-glycans of different individuals (Fig. 3b). The A antigen showed highest levels of expression, presented on 10-63% of all O-glycans in blood group A donors, while blood group B antigen was only expressed on 0–32% of all O-glycans in blood group B donors (Fig. 3b). It was not possible to determine the relative abundance of the H antigen on the N or O-linked glycans, because of the possibility of isomeric Lewis X/Lewis A antigen structures.

### ABO and the microbiome

Differential abundances of A and B glycan expression were seen depending on CST and pregnancy outcome (Fig. 3c). The highest abundance was type A on the O-linked glycans, and this was associated with *L. crispatus* dominance, and women delivering at term. In contrast, lower abundances of the type B were seen, and most women expressing the B antigen delivered preterm, regardless of CST. Bacterial binding of key bacterial species known to be present in the vagina (Supplementary Table 5) to a set of blood group glycan probes (Supplementary Table 6) was performed using glycan microarrays (Fig. 3d, e). Differential bacterial binding signals to blood group A type 2, B type 1, B type 2, H type 1 and H type 2 glycans were seen for *L. crispatus 1398, L. iners 13335, S. agalactiae* 776, and *G. vaginalis 540* strains at pH4 (Fig. 3e). Reduced binding was observed at pH7, except for the pathogenic species *L. iners, S. agalactiae*, and *G. vaginalis* to the H type 1 glycan. However, binding to blood group A on the type 1 backbone was consistently poor across all bacteria from the lower reproductive tract tested under various conditions. Our data show that bacteria from the vaginal microbiota can bind to blood group A, B, H glycans presented on glycan microarrays. These interactions could be part of the mechanism modulating bacterial composition in the female lower reproductive tract.

**Fig. 4:**
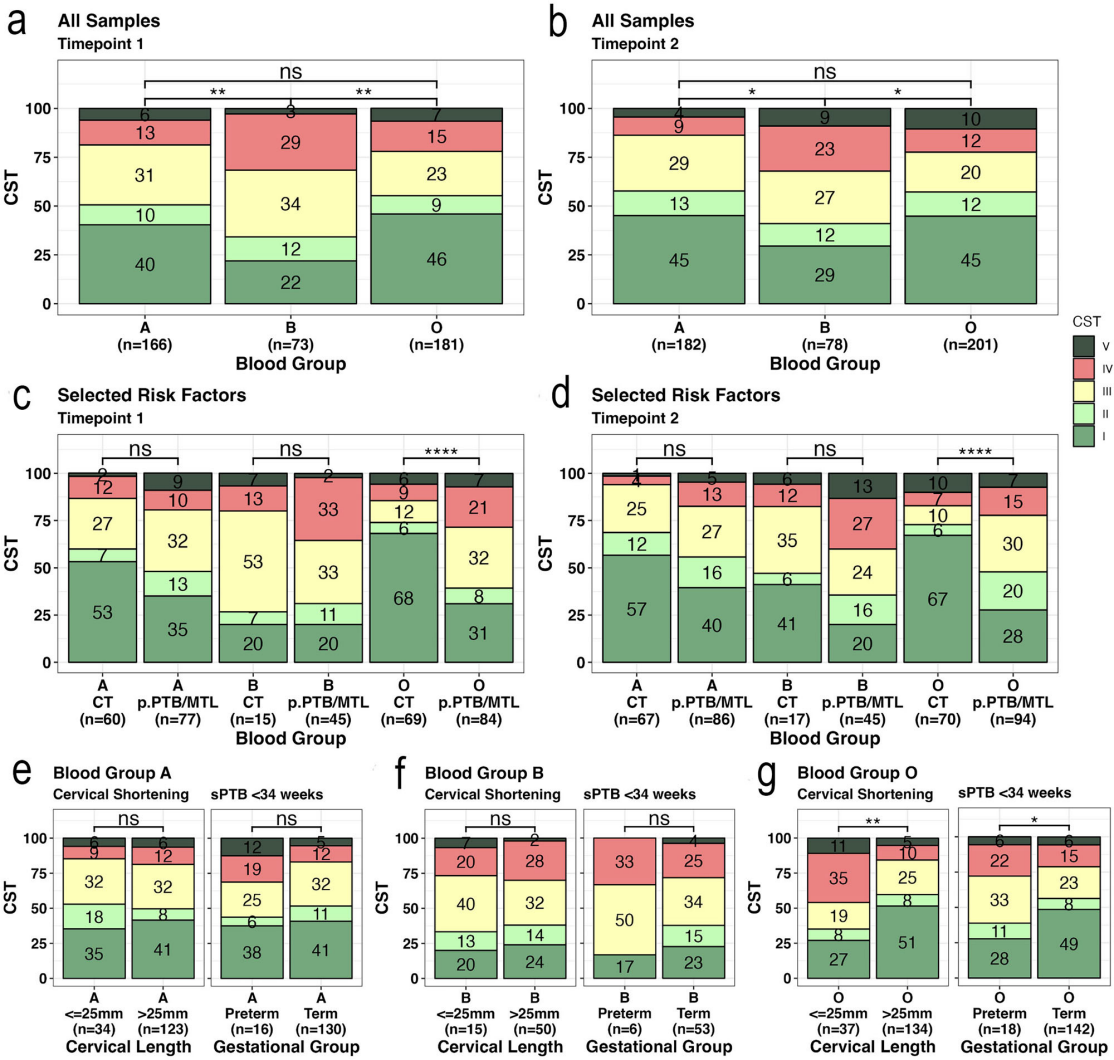
Vaginal Microbiome Community State Types (CSTs) according to blood group in women at risk of preterm birth. **a** Proportions of CST I-V in women defined as being at risk of preterm birth by ABO blood group phenotype at timepoint 1 (12+0–15+6), **b** and at timepoint 2 (20+0–23+6). **c** Proportions of CST1-V in women categorised by risk factors of previous cervical treatment (CT) or previous preterm birth of mid-trimester loss (pPTB/ MTL) and by ABO blood group phenotype at timepoint 1 (12^+0^–15^+6^) and at (**d**) timepoint 2 (20^+0^-23^+6^). Proportions of CST I-V at timepoint 1 (12 + 0-15 + 6) in women categorised by outcomes of cervical length and pregnancy outcome in women with blood group (**e**) A, (**f**) B and (**g**) O. Comparisons made using Chi-squared and adjusted for multiple comparisons. *P* value significance: *<0.05, **<0.01, ***<0.001, ****<0.0001.

Vaginal microbial composition was analysed from women at risk of preterm birth in early (timepoint 1) and mid-pregnancy (timepoint 2). Higher proportions of CST I were seen in blood group A and O women compared to blood group B (timepoint 1: vs A *p* < 0.01, vs. O *p* < 0.01; timepoint 2: vs A *p* = 0.03, vs. O *p* = 0.03), with enrichment for CSTs III and IV seen in blood group B (Fig. 4a, b). When CSTs were compared according to risk factors for sPTB, there was no significant difference between women with cervical treatment and those with a previous PTB or MTL in blood groups A and B at either time point (Fig. 4c, d). In women with blood group O and a previous sPTB or MTL, the proportion of CST I was lower than those with a history of cervical treatment (timepoint 1: 31% vs 68% *p* < 0.0001, timepoint 2: 28% vs 67% *p* < 0.0001) (Fig. 4c, d). Additionally, there were higher proportions of CST III and IV in women with previous PTB or MTL compared to previous cervical treatment in women of blood group O (Fig. 4c, d). We concluded that regardless of risk factor, in early pregnancy, women of blood group B are more likely to have adverse vaginal microbial communities (CST III/IV), and women with blood group A are more likely to show *L. crispatus* dominance. In contrast, women of blood group O maintain a favourable microbial environment in the context of previous cervical treatment, but in those who have experienced a previous PTB or MTL, there is a high chance of *L. crispatus* depletion.

The association between vaginal microbial composition, blood group and outcomes in the index pregnancy was assessed. In blood group A, there were no differences in microbial composition in women who experienced cervical shortening or sPTB <34 weeks compared to those who did not (Fig. 4e), implying that microbiome state is less likely aetiological factor in these women. In blood group B, there were high proportions of CST III and IV, low proportions of CST I, and no differences based on pregnancy outcome (Fig. 4f), and we concluded that adverse microbial composition alone is not sufficient to cause sPTB in these women. Women of blood group O who experienced cervical shortening and/or sPTB were significantly less likely to be CST I (*p* < 0.01 and *p* < 0.05) and had higher proportions of CST IV (Fig. 4g), indicating that adverse host responses to the microbial composition are a key aetiological factor.

### ABO and inflammation at the cervicovaginal interface and PTB

**Fig. 5:**
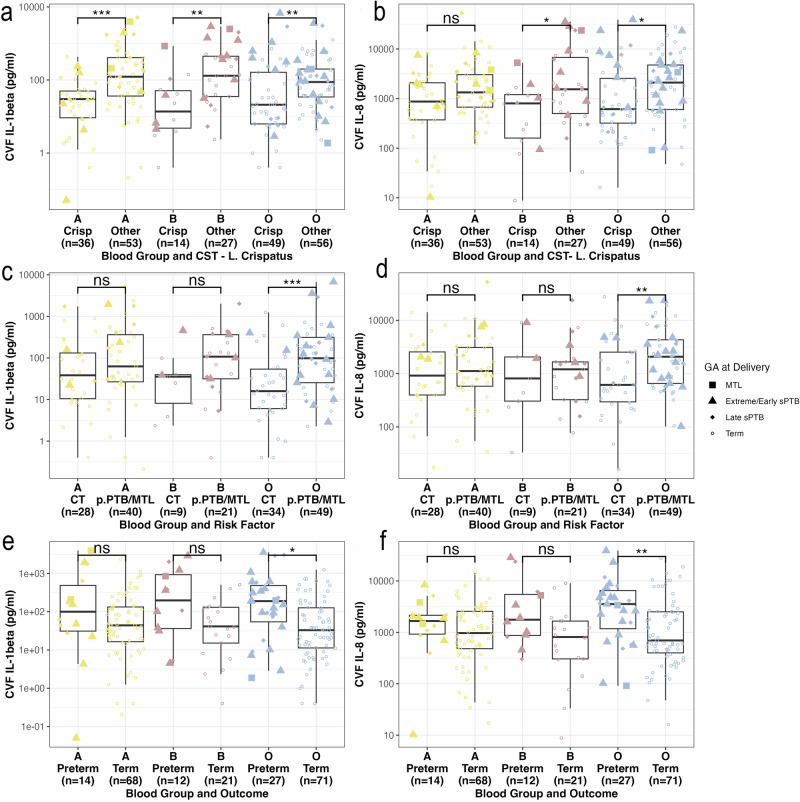
Cervicovaginal Fluid (CVF) cytokine concentrations by ABO blood group phenotype at timepoint 2 (20 + ^0^-23 + ^6^). **a** CVF IL-1$\beta$ (pg/ml) and community state type (CST) (*n* = 219). **b** CVF IL-8 (pg/ml) and CST (*n* = 219). **c** CVF IL-8 (pg/ml) and risk factor (*n* = 180). **d** CVF IL-8 (pg/ml) and risk factor (*n* = 180). **e** CVF IL-1$\beta$ (pg/ml) and sPTB (iPTB excluded) (*n* = 202). **f** CVF IL-8(pg/ml) and sPTB (iPTB excluded) (*n* = 202). Wilcoxon Signed-Rank Test adjusted for multiple comparisons (*p* < 0.05*, *p* < 0.01**, *p* < 0.001***, *p* < 0.0001****).

IL-1β concentrations were higher in women with CSTs not dominated by *L. crispatus* in all blood groups (A: *p* < 0.001, B: *p* < 0.01, O: *p* < 0.01) (Fig. 5a). IL-8 concentrations were significantly elevated in non-*L. crispatus* CSTs in women of blood groups B and O, but not A (B: *p* < 0.05, O: *p* < 0.05), demonstrating stronger inflammatory phenotypes in these blood groups, and a differential effect of the A phenotype on IL-1β production compared to IL-8 (Fig. 5b). At timepoint 1, the same responses were seen for IL-1β but to a lesser degree, and there were no significant relationships for IL-8 by CST, supporting an increase in inflammatory response to microbial composition across gestational age (Supplementary Fig. 2a, b). Comparison of cytokine concentrations according to risk factor demonstrated that in women of blood group O, IL-1β and IL-8 were significantly higher in those with a history of previous sPTB or MTL compared with previous cervical treatment (*p* < 0.001 and *p* < 0.01 respectively), a relationship not seen in the other blood groups (Fig. 5c, d). This is likely to be reflective of the reduced prevalence of CST I seen in blood O women with previous sPTB or MTL. The same relationships were seen in early pregnancy at timepoint 1 (Supplementary Fig. 2c, d), demonstrating a pro-inflammatory phenotype in blood group O women with previous sPTB or MTL.

In blood group A, there was no association between IL-1β or IL-8 concentrations and pregnancy outcome, whereas in blood group O, concentrations are significantly higher in those who deliver preterm compared to term (IL-1β *p* = 0.05, IL-8 *p* = 0.01) at timepoint 2 (Fig. 5e, f). In blood group B women, there was a higher median concentration of cytokines in women who delivered preterm at timepoint 2, but this was not statistically significant. Only IL-1β in blood group O was significantly higher in women who delivered preterm in early pregnancy at timepoint 1 (*p* = 0.04) (Supplementary Fig. 2e, f). These data are consistent with local inflammation driving preterm birth, particularly in blood group O women.

**Fig. 6:**
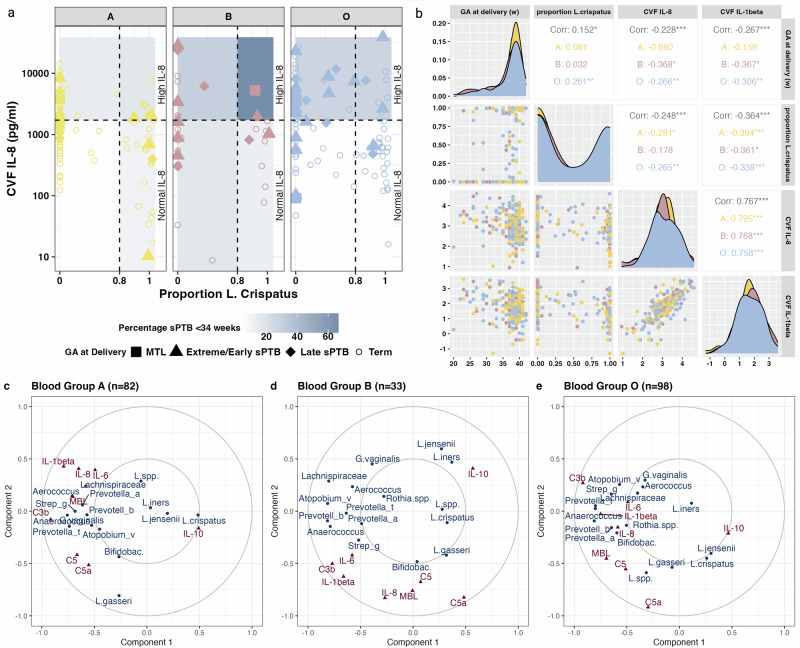
Associations between cervicovaginal immune mediators, vaginal microbiota and ABO blood group at timepoint 2 (20 + ^0^-23 + ^6^). **a** CVF IL-8 – classified as normal or high (according to tertile) by ABO blood group and *Lactobacillus Crispatus* dominance (defined as 80% relative abundance) with the shading in each quadrant indicating the percentage of sPTB <34 weeks gestational age at is shown in. **b** Correlation plots for gestational age at delivery*, Lactobacillus crispatus* proportion, CVF IL-8 and IL-1β concentrations for blood groups A (yellow), B (red) and O (blue) are shown in. (**c-e)**, Canonical Correlation Analysis between 20 dominant bacterial taxa across samples (shown in blue) and CVF immune analytes (shown in red). Objects that are close together are correlated with each other. Distance from the centre represents strength of association. **c** Blood group A (*n* = 82). **d** blood group B (*n* = 33). **e** Blood Group O (*n* = 98). List of 20 dominant taxa: *Lactobacillus crispatus, Lactobacillus iners, Gardnerella vaginalis, Lactobacillus gasseri, Lactobacillus jensenii, Bifidobacterium, Atopobium vaginae, Streptococcus, Lactobacillus spp., Prevotella timonensis, Aerococcus, Lachnospiraceae, Prevotella bivia, Prevotella amnii, Anaerococcus, Rothia, Finegoldia, Peptoniphilus, Atopobium rimae, Fusobacterium*.

In women of blood group A, the rate of sPTB <34 weeks was similar for women regardless of IL-8 and *L. crispatus* proportion (Fig. 6a). In women of blood group B, sPTB <34 weeks was lowest in those with normal IL-8 who were dominant (13%) or depleted (17%) for *L. crispatus*, and highest in those with high IL-8 who were dominant for *L. crispatus* (67%). In blood group O women, the highest rate of sPTB <34 weeks was seen in those who were both high in IL-8 and deplete of *L. crispatus* (31%), with the number of preterm births in this quadrant being 4 times that of any of the others (Fig. 6a). In blood group O women there was a significant correlation between gestational age at delivery, proportion of *L. crispatus* and IL-8 and IL-1β (Fig. 6b). In blood group B women, there were significant correlations between gestational age at delivery and IL-8 and IL-1β, but not proportion of *L. crispatus*. In blood group A women, whilst there were significant correlations between the proportion of *L. crispatus* and IL-8 and IL-1β, none of these were correlated with gestational age at delivery (Fig. 6b).

Canonical correlation analysis of the 20 dominant taxa across samples and CVF immune analytes according to blood group showed association between *L. crispatus* and the anti-inflammatory cytokine IL-10 in blood group A (Fig. 6c). No correlation between diverse bacterial taxa and pro-inflammatory immune analytes was seen in women of blood group B (Fig. 6d), suggesting an alternative driver of this inflammatory response. In blood group O, correlations between diverse taxa pro-inflammatory analytes were observed (Fig. 6e). Women with blood group O delivering preterm are characterised by an adverse vaginal microbiota and high levels of inflammation. In blood group A, the same microbiota does not evoke a strong inflammatory response and thus does not result in early delivery. In blood group B, there is an inflammatory phenotype associated with earlier delivery, but this is not driven by an adverse vaginal microbiota. These results may reflect differing aetiological pathways in women delivering preterm on the basis of blood group.

## Discussion

Despite microbial—immune driven sPTB being considered to be the most common causal factor for sPTB, there is a lack of the mechanistic understanding required for the development of new and individualised preventative therapies. This study provides evidence for a role for cervicovaginal ABO(H) glycans and the ABO blood group phenotype in influencing cervicovaginal microbial composition, immune *milieu* and risk of sPTB. Although ABO glycan binding of microbiota in the context of the vaginal microbiome has not previously been described, reports exist of *lactobacilli* species, including *Lactobacillu*s *crispatus* binding strongly to type A glycans, and weakly to type B in the context of gut microbiome health^29,32^. Our glycan array data showed that *L. crispatus and L.iners* bind to A type 2 at pH4; however, *L. crispatus* was the only species able to bind to the A glycan at pH7. In contrast, only the pathogenic species *L. iners, S. agalactiae* and *G.vaginalis* showed binding at pH7 and this was to the H type 2 glycan. This difference in A and O(H) glycan-microbe interaction may explain why women with the A blood group phenotype are more likely to have *L. crispatus* dominance, have lower concentrations of pro-inflammatory cytokines, and are more likely to deliver at term compared to women of the O phenotype. Preterm birth that occurs in blood group A phenotype is therefore more likely to be due to a mechanism unrelated to microbe-induced local inflammation compared to other blood group phenotypes.

This study has demonstrated an increased risk of sPTB in women of blood group B both in a general maternity population, and in a population at-risk of preterm birth. We also saw a clear association with a vaginal composition depleted in *L. crispatus*, and a vaginal microbiome with high bacterial diversity. We demonstrated B glycan expression on N and O-linked glycans predominantly in women who delivered preterm. Our glycan array data showed the strongest binding of *S. agalactiae* with a type 1 B glycan probe which may explain why women of blood group B are more likely to be colonised with Group B *Streptococcus* than women of blood group O and A^24^. The most striking increase in risk of sPTB in the blood group B phenotype was seen in women who had a history of previous cervical treatment. Cervical treatment is performed for women with cervical intraepithelial neoplasia (CIN). CIN and cervical cancer are caused by oncogenic Human Papilloma Virus (HPV) strains, mainly HPV-16, 18, 31, 33 and 45^26^. The presence and persistence of HPV, and the development of high-grade CIN/cancer is associated with high diversity and the deviation away from *Lactobacillus* dominance^33,34^. The possible co-existence of HPV and an adverse microbiome in women of blood group B may explain the increased risk of cervical shortening and sPTB in women with a history of cervical treatment either through inflammation secondary to microbial composition or mechanical damage following treatment. Local IL-1β and IL-8 concentrations are elevated in CIN, and this inflammation persists at 6-months post-excisional treatment despite disease and HPV clearance^35^. This may explain why there was a pro-inflammatory response in women of blood group B who delivered preterm, yet no clear association between bacterial taxa and pro-inflammatory mediators. Alternatively, HPV carriage may be driving this inflammation, as between 15-58% of women of childbearing age are HPV positive^36^. Although we do not have information on HPV carriage in this study population, this will be a focus of our future work.

A and B glycans have been shown to be expressed in the squamous epithelium of the cervix, whereas H antigens are more highly expressed in endocervical epithelium^37^. There is a loss of the A and H antigen in premalignant and malignant lesions of the cervix^38^. This differential expression of ABO(H) glycans to favour B glycan expression over A or H, or changes in concentrations of anti-A antibodies may also explain the predominance of adverse pregnancy outcomes in women of blood group B who have had previous cervical treatment for CIN. Our future work will aim to develop an understanding between cervical stroma, ABO blood group secretion and the various sPTB phenotypes, like those who develop cervical shortening in the context of previous excisional treatment to the cervix in women with premalignant lesions.

Our study demonstrates that women with a history of sPTB of blood group O had higher rates of early sPTB compared to women of blood group A and shows a correlation between *Lactobacillus* spp. depletion and a pro-inflammatory *milieu*. It is plausible that anti-A and anti-B natural antibodies play a role, as they can be detected in cervical mucus of blood group O, even when absent in serum^39^. Natural antibodies to A and B are mainly IgM or less frequently IgG and IgA^40^, and are generally not antigen specific^41^. They do offer polyreactivity on a wide range of bacteria and viruses^42,43^, and can be potent inducers of complement activation^40,44^. In contrast, anti-O(H) is thought not to be influenced by microbial stimulation^41^. This may explain why women who delivered preterm in blood group O had a clearer association with microbial composition and a pro-inflammatory immune *milieu*, than women of blood group A and B. Our future work will explore the presence and role of ABO antibodies in CVF.

The effect size of the influence of ABO blood phenotype on sPTB risk is not as strong as other factors such as race or risk factors cervical treatment and prior preterm birth. This implies that the interaction between ABO, microbiome and inflammation is only one element of a sequence of events that lead to preterm birth. One limitation of this study is that we did not have known secretor status for the study population which is influenced by race, and we did not have known concentrations of secreted blood group glycans and their antibodies, which may also impact on the relative risk. Nevertheless, we believe this study provides data to support the potential use of glycan therapies to target host–microbe interactions for preterm birth prevention, especially with the added risk stratification of ABO status. Strategies are in development targeting host–microbe interactions relevant to the reproductive tract. Examples include host glycan mimicry using high-affinity synthetic glycans resembling host glycans recognised by *N. gonorrhoeae*, and targeting bacterial glycans like *E.coli* and Group B *Streptococcus* with monoclonal antibodies and vaccines^45^.

Our data provide a novel mechanism of a genetically determined predisposition to, and protection against, sPTB in a large UK multi-ethnic population. Collectively, our findings indicate a role of ABO(H) glycans and the ABO phenotype in influencing microbial composition, immune *milieu* and pregnancy outcome, and serve as a novel predictive and therapeutic target for preterm birth prevention.

## Methods

### Study design and population

Observational data from 85,044 anonymised maternities from two inner London hospitals in the UK, Queen Charlottes and Chelsea Hospital and St Mary’s Hospital, Imperial College Healthcare NHS Trust, between 2014 and 2023. These women were identified using electronic healthcare records and anonymised data was analysed using electronic records via Imperial Clinical Analytics Research and Evaluation (iCARE) environment (Fig. 1a, b).

Data on women who were defined as being ‘at-risk’ of preterm birth was collected from women participating in the Vaginal Microbiome and Metabonome in Pregnancy (VMET-2) Research study. A total of 2084 women were prospectively recruited from Preterm Birth Prevention Clinics across five participating sites (Queen Charlottes and Chelsea Hospital, St Mary’s Hospital, Chelsea and Westminster Hospital, and University College Hospital (all in London), and the Royal Infirmary Edinburgh Hospital) between 2014 and 2023 (Fig. 1a–c). Complete metadata was collected from MedSciNet© (CSAM, Oslo, Norway) and electronic patient records (Cerner, EPR Solutions, North Kansas City, MO, USA). Women were defined as being at risk of preterm birth if they had a history of cervical excisional treatment for pre-cancerous cells, a previous preterm birth or mid-trimester loss, or other risk factors such as previous caesarean section at full cervical dilation or congenital uterine anomaly. The most common risk factors of women seen in preterm birth prevention clinics are previous excisional cervical treatment, previous spontaneous preterm birth < 34 weeks, including mid-trimester loss < 24 weeks of pregnancy. Of this high-risk population, vaginal microbiome sampling was performed in 596 women, and both microbiome and immune mediator analyses were performed in 314 women. A subset of 42 of women donated Softcup® samples that were used for glycomics analyses.

### Outcomes

The primary outcome was sPTB <37 weeks of gestational age. PTB was defined as either sPTB either following spontaneous onset of labour with cervical dilatation, or prelabour premature rupture of membranes (PPROM), or iPTB following a delivery initiated by care providers in response to maternal or foetal complications of pregnancy. Secondary outcomes were sPTB <34 weeks of gestational age, cervical length (CL) as measured by transvaginal ultrasound scan (TVUS). ABO blood group and information on self-reported ethnicity were obtained from electronic records.

### Sample collection

In the high-risk population, women attending the preterm birth prevention clinics were given the opportunity to be recruited to the VMET-2 study. Consenting women provided vaginal swabs at timepoint 1 (12 + 0 – 15 + 6 weeks) and at timepoint 2 (20 + 0 - 23 + 6 weeks) unless delivery occurred before. Swabs were inserted into the mid-high region of the vaginal canal prior to performing an ultrasound scan. After collection, the BBL™ culture swab™ MaxV Liquid Amies swabs (Becton Dickinson, Oxford, UK) were placed on ice immediately and stored at –80 °C. Cervical length was measured at each visit by transvaginal ultrasound as part of routine care. Metadata including ABO blood group, maternal age, BMI, self-reported ethnicity, past obstetric history and PTB prevention interventions, were collected at each visit and stored securely. Women were followed up to determine pregnancy outcome. In a subset, CVF was collected using a disposable menstrual disc (Softcup®) that was placed past the vaginal canal for 20 min. After its removal, contents were resuspended in a 1:5 weight:volume ratio with sterile phosphate buffer saline (PBS) and stored at −80 °C until use.

### Microbiome composition

Swabs containing cervicovaginal fluid (CVF) were thawed and supernatant was retrieved by pressurising the sponge of the swab with a sterile syringe in preparation for DNA extraction, and confirmation of DNA integrity by polymerase chain reaction (PCR) amplification was performed as previously described^46^. The V1-V2 hypervariable regions of the 16S rRNA genes were amplified for sequencing, using forward and reverse primers. The forward primer set (28f-/YM) consisted of a mixture of the following primers mixed to a 4:1:1:1 ratio; 28F-Borrellia GAGTTTGATCCTGGCTTAG; 28F-Chlorlex GAATTTGATCTTGGTTCAG; 28F-Bifido GGGTTCGATTCTGGCTCAG; 28FYM GAGTTTGATCNTGGCTCAG. This mixed formulation of the 27 F forward primer (27F-YM) has been shown to maintain the rRNA gene ratio of *Lactobacillus* spp. to *Gardnerella*^47^. The reverse primer consisted of 388 R GCTGCCTCCCGTAGGAGT. Sequencing was then performed using an Illumina MiSeq platform, at RTL Genomics (Lubbock, TX, USA). Sequence data was then analysed, aligned and classified to operational taxonomic units (OTUs) (phylum to genus level) using Ribosomal Database Project reference sequence files and species-level taxonomies using USEARCH; or to amplicon sequence variant (ASV)- level using the QIIME2 bioinformatics pipeline (version 2022.2.1)^48^ with taxonomic assignments mapped to the STIRRUPS database^49^. Community state type (CST) classifications were generated from counts summed to species-level or the next highest taxa-level as per naming conventions required by the Vaginal Community State Type Nearest Centroid Classifier (VALENCIA) algorithm^50^. Based on VALENCIA CST assignments, samples were grouped into five main CSTs: 1) CST I (*L. crispatus* dominated); 2) CST II (*L. gasseri* dominated); 3) CST III (*L. iners* dominated); 4) CST IV (*Gardnerella vaginalis* dominated); and 5) CST V (*L. jensenii* dominated). For canonical correlation analysis the 20 most abundant taxa were identified across all samples using total read counts for all taxons.

### Immune profiling

CVF was extracted from thawed swabs by pressurising the sponge of the swab with a sterile syringe until dry and centrifuged at 4500*g* for 10 min at 4 °C to collect the supernatant, which was stored at −20 °C. Cytokines and complement proteins were quantified using Luminex ® immunoassays following manufacturer’s instructions, including the concentration of cytokines IL-1β, IL-6, IL-10 (Luminex Human Discovery Assay 3-plex, neat), IL-8 (Luminex Human Discovery Assay 1-plex, 1:10 dilution using Calibrator Diluent RD6-52) (R&D Systems) and complement proteins MBL, C5 C5a (Human Complement Magnetic Bead Panel 1, HCMP1MAG-19K) and C3b (Human Complement Magnetic Bead Panel 2, HCMP2MAG-19K (Milliplex®) and results expressed as nanograms or picograms/ml. All samples were analysed using a 96-well plate, acquired in a MagPix®analyser/Bio-Plex 200 system and analysed with xPONENT ® software. Samples were run in duplicates, with inter and intraplate controls.

### ABO glycan profiles

For analyses of glycan profiles, CVF was collected using a menstrual cup (Softcup®) by placing it against the cervix for 20 min. After removal, material was retrieved by repeated pipetting of phosphate buffer saline (PBS) to resuspend at a 1:5 weight: volume ratio within 30 min of collection and stored at −80 °C. Glycomic sample processing was done following the protocol detailed previously^30^. Briefly, CVF samples were sonicated in 25 mM Tris, 150 mM NaCl, 5 mM EDTA, and 1% CHAPS, pH 7.4, dialysed in dialysis cassettes, reduced by DTT, carboxymethylated by IAA, and digested by trypsin. N-glycans were released by PNGase F, separated from O-glycopeptides by C18 Sep-Pak chromatography and were then permethylated following the NaOH procedure^51^. O-glycans were released from glycopeptides through reductive elimination: four hundred microliters of 0.1 M KOH containing potassium borohydride (54 mg/mL) was added to dried samples and incubated at 45 °C for 14–16 h. The reaction was terminated by adding a few drops of 5% (v/v) acetic acid followed by purification with Dowex 1-X8 desalting column. Excess borates in the samples were subsequently removed by co-evaporating with 10% (v/v) acetic acid in methanol under a stream of nitrogen at room temperature. The purified native O-glycans were then permethylated following the NaOH procedure. The methylated glycans were dissolved in 10 µl methanol. An aliquot of 1 µl of sample was mixed with 1 µl of 10 mg/ml DABP matrix in 75% ACN. The mixture was spotted on a MALDI plate for MALDI-TOF-MS and MS/MS analysis. Data were analysed using Data ExplorerTM version 4.6 from AB Sciex, Glycoworkbench72 and MALDIquant73. The glycomic data were annotated based on monosaccharide composition derived from the molecular ion m/z value, knowledge of glycan biosynthetic pathways, the isotopic peak cluster patterns, the glycosylation patterns in the low and medium mass range, and MS/MS fragmentation.

### Bacterial culture and preparation for glycan array binding studies

Bacterial isolates were streaked onto their corresponding agar (Supplementary Table 5) and incubated anaerobically (37 °C; gas composition 10% CO_2_, 10% hydrogen, 80% nitrogen; 70% humidity). The culture was used to inoculate 10 ml of degassed broth (Supplementary Table 5) which was incubated anaerobically overnight. The overnight culture was then used to inoculate fresh degassed broth which was incubated anaerobically until the optical density (600 nm) reached 1 (±0.05).

All bacteria were prepared live and fluorescently labelled. Aliquots (2 ml) of culture were centrifuged at 5000 g for 10 min and the pellet was washed twice using cold, degassed Hanks Balanced Salt Solution (HBSS; Gibco). The culture was then stained by incubating the cells in 10 μM CellTrace™ Far Red (Molecular Probes) in PBS for 1 h on a rotating wheel protected from light. The culture was washed twice using HBSS (as above). The prepared culture was resuspended in HBSS and kept on ice and protected from light until further use.

### Preparation and analysis of AEAB-terminating glycan probes

AEAB-terminating probes were prepared from reducing glycans by reductive amination. Neutral blood group terminating glycans from glycan probes #3158, #3159, #3161, #3162, #3330 and #3164 were purchased from Elicityl (Crolles, France) (Supplementary Table 6). Preparation of the fluorescent reagent AEAB and conjugation with reducing sugars by reductive amination were performed as previously described^52^. For purification of the conjugation product, a Sep-Pak Aminopropyl cartridge (Waters, Wilmslow, England) was used with elution by acetonitrile /H_2_O for the neutral sugars and acetonitrile /0.05 M ammonium acetate for the sialylated sugars. Further purification was carried out by HPLC using Amide column (XBridge, Waters). The gradient elution was acetonitrile /H_2_O at a flow rate of 1 ml/min with detection by UV 330 nm.

The micro-scale dot assay on TLC plates was performed as described elsewhere^53^. Briefly, hexose-containing probes were dissolved in H_2_O (approx. 0.5–1.0 mg/ml), before 1 μl was deposited on silica gel TLC plate. Standard solutions (Gal in H_2_O) were also deposited alongside the sample. The plate was sprayed with orcinol/H_2_SO_4_ reagent before heating in an oven at 105 ^o^C to develop colour. Quantitation was carried out by scanning at 550 nm on a CAMAG TLC scanner (Omicron Research, Hungerford, England).

MS analysis of most amino-terminating probes was carried out by electrospray mass spectrometer on a Waters (Manchester, UK) Q-TOF-type mass spectrometer SYNAPT-G2 in either positive- or negative ion mode. Cone voltage was at 50 eV or 80 eV and capillary voltage at 3 kV. Source temperature was at 80 ^o^C and the desolvation temperature at 150 ^o^C. A scan rate of 1.5 sec/scan was used and the acquired spectra were summed for presentation. For analysis, glycan probes were dissolved in H_2_O typically at a concentration of 10-20 pmol/μl, of which 1 μl was injected via a HPLC injector. Solvent acetonitrile/2 mM ammonium bicarbonate 1:1 was delivered by a HPLC pump (Waters) at a flow rate of 10 μl/min. Selected probes were also analysed by MALDI-MS on an Axima MALDI Resonance mass spectrometer with a QIT-TOF configuration (Shimadzu). A nitrogen laser was used to irradiate samples at 337 nm, with an average of 100-200 shots accumulated. A matrix solution (0.5 μl) of 2,5-dihydroxybenzoic acid (20 mg/ml) in a mixture of methanol/water (1:1) was deposited on the sample target before application of the sample solution (0.5 μl).

### Glycan microarray preparation and analyses

The generation of covalent sequence-defined glycan arrays on NHS-functionalised glass slides (Nexterion H) was performed essentially as described previously^54,55^. Two oligosaccharide subarrays were generated. Details of the preparation of the microarrays and the methods used for binding assays and data analysis are in accordance with MIRAGE (Minimum Information Required for A Glycomics Experiment) guidelines for reporting of glycan microarray-based data (Supplementary Information)^56^.

Microarray analyses were performed essentially as previously described^57^ at ambient temperature. No blocking was used for overlay assays on covalent glycan array glass slides. Details of the antibodies used for glycan microarray quality control are summarised in the MIRAGE document in Supplementary Information.

Microarray analyses of fluorescently labelled bacteria were performed as follows. Fluorescently labelled bacterial cultures at OD = 1 were resuspended in binding buffer (10 mM Hepes at pH 7.5 or pH 4, 150 mM NaCl, and 5 mM CaCl_2_) and were used for overlays on glycan microarrays. 100 µL of bacterial suspension was applied to the incubation chamber with the microarrays, incubated for 1 h at room temperature under mild agitation on an oscillating platform and washed three times with binding buffer followed by two washes with HPLC grade water to remove salts from the array. Slides were dried under a mild nitrogen flow and scanned for quantitation as described below.

Images of fluorescently bound antibodies and bacteria were acquired with a GenePix 4300 A scanner from Molecular Devices, quantified using GenePix software and analysed with CarbArryART^58^. In glycan microarray binding analyses each assay was performed with four technical replicates and mean (MFI) and standard deviation (SD) values of quadruplicates data can be found in Supplementary Data 1. Heatmaps of processed data were generated using excel.

### Statistical analyses

Statistical analyses were performed using R (version 4.2.3)^59^. Summary statistics and demographics were derived and stratified according to ABO blood group phenotype, risk factor for sPTB and self-reported ethnicity. Statistical significance was assumed at *p* < 0.05. Univariable comparisons of categorical data were completed using Chi-square test or Fisher’s exact test. Continuous data were assessed for normality using Kolmorgorov–Smirnov tests and presented as mean (±standard deviation) or median (±inter-quartile range). Either t-tests or Mann–Whitney *U* tests were used for comparisons as appropriate. All tests and *p* values were adjusted for multiple comparisons. Logistical regression analysis was completed with sPTB <37 weeks as the outcome variable, with ABO blood group, self-reported ethnicity, history of previous PTB or MTL or history of cervical treatment included as dependent variables.

### Ethics and reporting

Data from the anonymised general maternity population from iCARE was approved by the NIHR Imperial BRC Data Access and Prioritisation Committee (under HRA database approval (20HH6447)). The study of women at risk of preterm birth was approved by the National Health Service, National Research Ethics Committee in Stanmore, London (REC 14/LO/03238), and these participants provided written informed consent. This research was performed in accordance with the Declaration of Helsinki. Inclusion criteria were previous excisional cervical treatment, previous sPTB or mid-trimester loss, fully dilated caesarean section or a history of uterine anomaly. Exclusion criteria included women under the age of 18, women with HIV/ Hepatitis B/C, vaginal intercourse or bleeding within 72 h of sample collection.

## Supplementary information

Supplementary information

Supplementary information

## Data Availability

Data supporting this study are available in this article and in the Supplementary Information files. All sequence data is available on the ENA database browser under accession codes PRJEB11895, PRJEB12577, PRJEB30642, PRJEB41427, PRJEB83084 and PRJEB89979. Data from the general population cannot be shared publicly because of data protection and governance restrictions. Requests to access data within the ICHT ICARE Secure Data Environment can be made via imperial.dataaccessrequest@nhs.net referring to the title of this paper.

## Code availability

Key analysis code and processed datasets are publicly available via the GitHub repository: https://github.com/katiemountain0305/Mountain_2025_ABO_microbiome. As the data from the general obstetric population cannot be publicly shared, illustrative code is provided. Data analyses were performed using R (version 4.2.3).

## References

[1] Ohuma, E. O. et al. National, regional, and global estimates of preterm birth in 2020, with trends from 2010: a systematic analysis. *Lancet* **402**, 1261–1271 (2023). 37805217 10.1016/S0140-6736(23)00878-4

[2] Perin, J. et al. Global, regional, and national causes of under-5 mortality in 2000-19: an updated systematic analysis with implications for the Sustainable Development Goals. *Lancet Child Adolesc. Health* **6**, 106–115 (2022). 34800370 10.1016/S2352-4642(21)00311-4 PMC8786667

[3] Goldenberg, R. L., Culhane, J. F., Iams, J. D. & Romero, R. Epidemiology and causes of preterm birth. *Lancet* **371**, 75–84 (2008). 18177778 10.1016/S0140-6736(08)60074-4 PMC7134569

[4] Huang, C. et al. Meta-analysis reveals the vaginal microbiome is a better predictor of earlier than later preterm birth. *BMC Biol.* **21**, 199 (2023). 37743497 10.1186/s12915-023-01702-2 PMC10518966

[5] Manuck, T. A. et al. Preterm neonatal morbidity and mortality by gestational age: a contemporary cohort. *Am. J. Obstet. Gynecol.* **215**, 103 e1– e14 (2016). 26772790 10.1016/j.ajog.2016.01.004 PMC4921282

[6] Golob, J. L. et al. Microbiome preterm birth DREAM challenge: crowdsourcing machine learning approaches to advance preterm birth research. *Cell Rep. Med.* **5**, 101350 (2024). 38134931 10.1016/j.xcrm.2023.101350 PMC10829755

[7] Kindinger, L. M. et al. The interaction between vaginal microbiota, cervical length, and vaginal progesterone treatment for preterm birth risk. *Microbiome* **5**, 6 (2017). 28103952 10.1186/s40168-016-0223-9 PMC5244550

[8] Chan, D. et al. Microbial-driven preterm labour involves crosstalk between the innate and adaptive immune response. *Nat. Commun.* **13**, 975 (2022). 35190561 10.1038/s41467-022-28620-1 PMC8861006

[9] Kindinger, L. M. et al. Relationship between vaginal microbial dysbiosis, inflammation, and pregnancy outcomes in cervical cerclage. *Sci. Transl. Med.* **8**, 350ra102 (2016). 27488896 10.1126/scitranslmed.aag1026

[10] Bayar, E., Bennett, P. R., Chan, D., Sykes, L. & MacIntyre, D. A. The pregnancy microbiome and preterm birth. *Semin. Immunopathol.* **42**, 487–499 (2020). 32797272 10.1007/s00281-020-00817-w PMC7508933

[11] Ravel, J. et al. Vaginal microbiome of reproductive-age women. *Proc. Natl Acad. Sci. USA* **108**, 4680–4687 (2011). 20534435 10.1073/pnas.1002611107 PMC3063603

[12] Petricevic, L. et al. Characterisation of the vaginal Lactobacillus microbiota associated with preterm delivery. *Sci. Rep.* **4**, 5136 (2014). 24875844 10.1038/srep05136 PMC4038809

[13] Tabatabaei, N. et al. Vaginal microbiome in early pregnancy and subsequent risk of spontaneous preterm birth: a case-control study. *BJOG* **126**, 349–358 (2019). 29791775 10.1111/1471-0528.15299

[14] Gudnadottir, U. et al. The vaginal microbiome and the risk of preterm birth: a systematic review and network meta-analysis. *Sci. Rep.* **12**, 7926 (2022). 35562576 10.1038/s41598-022-12007-9 PMC9106729

[15] Fettweis, J. M. et al. The vaginal microbiome and preterm birth. *Nat. Med* **25**, 1012–1021 (2019). 31142849 10.1038/s41591-019-0450-2 PMC6750801

[16] Dotz, V. & Wuhrer, M. Histo-blood group glycans in the context of personalized medicine. *Biochim Biophys. Acta* **1860**, 1596–1607 (2016). 26748235 10.1016/j.bbagen.2015.12.026 PMC7117023

[17] Jajosky, R. P. et al. ABO blood group antigens and differential glycan expression: Perspective on the evolution of common human enzyme deficiencies. *iScience* **26**, 105798 (2023). 36691627 10.1016/j.isci.2022.105798 PMC9860303

[18] Griffin, N. R. & Wells, M. Semiquantitative immunohistochemical studies of blood group antigen A, B, H, Le(a), Le(b)structures and Ii backbone chains in the normal human cervix and in cervical adenocarcinoma. *Histochem J* **25**, 228–241 (1993). 8473202 10.1007/BF00163819

[19] Navas, E. L. et al. Blood group antigen expression on vaginal and buccal epithelial cells and mucus in secretor and nonsecretor women. *J. Urol.* **149**, 1492–1498 (1993). 7684790 10.1016/s0022-5347(17)36425-x

[20] Cooling, L. Blood groups in infection and host susceptibility. *Clin. Microbiol. Rev.* **28**, 801–870 (2015). 26085552 10.1128/CMR.00109-14 PMC4475644

[21] Makivuokko, H. et al. Association between the ABO blood group and the human intestinal microbiota composition. *BMC Microbiol.* **12**, 94 (2012). 22672382 10.1186/1471-2180-12-94 PMC3485159

[22] Abegaz, S. B. Human ABO blood groups and their associations with different diseases. *Biomed. Res Int* **2021**, 6629060 (2021). 33564677 10.1155/2021/6629060 PMC7850852

[23] Foster, M. T. Jr. & Labrum, A. H. Relation of infection with Neisseria gonorrhoeae to ABO blood groups. *J. Infect. Dis.* **133**, 329–330 (1976). 1254989 10.1093/infdis/133.3.329

[24] Regan, J. A., Chao, S. & James, L. S. Maternal ABO blood group type B: a risk factor in the developement of neonatal group B streptococcal disease. *Pediatrics* **62**, 504–509 (1978). 362365

[25] Bianchi-Jassir, F. et al. Preterm birth associated with group B Streptococcus maternal colonization worldwide: systematic review and meta-analyses. *Clin. Infect. Dis.* **65**, S133–S142 (2017). 29117329 10.1093/cid/cix661 PMC5850429

[26] Vallely, L. M. et al. Adverse pregnancy and neonatal outcomes associated with Neisseria gonorrhoeae: systematic review and meta-analysis. *Sex. Transm. Infect.* **97**, 104–111 (2021). 33436505 10.1136/sextrans-2020-054653 PMC7892372

[27] Gampa, A., Engen, P. A., Shobar, R. & Mutlu, E. A. Relationships between gastrointestinal microbiota and blood group antigens. *Physiol. Genomics* **49**, 473–483 (2017). 28710295 10.1152/physiolgenomics.00043.2017 PMC5625272

[28] Kinoshita, H. et al. Cell surface glyceraldehyde-3-phosphate dehydrogenase (GAPDH) of Lactobacillus plantarum LA 318 recognizes human A and B blood group antigens. *Res. Microbiol.* **159**, 685–691 (2008). 18790050 10.1016/j.resmic.2008.07.005

[29] Uchida, H. et al. Lactobacilli binding human A-antigen expressed in intestinal mucosa. *Res. Microbiol.* **157**, 659–665 (2006). 16631357 10.1016/j.resmic.2006.03.001

[30] Wu, G. et al. N-glycosylation of cervicovaginal fluid reflects microbial community, immune activity, and pregnancy status. *Sci. Rep.* **12**, 16948 (2022). 36216861 10.1038/s41598-022-20608-7 PMC9551102

[31] Wu, G. et al. Glycomics of cervicovaginal fluid from women at risk of preterm birth reveals immuno-regulatory epitopes that are hallmarks of cancer and viral glycosylation. *Sci. Rep.* **14**, 20813 (2024). 39242814 10.1038/s41598-024-71950-x PMC11379862

[32] Uchida, H. et al. Lactic acid bacteria (LAB) bind to human B- or H-antigens expressed on intestinal mucosa. *Biosci. Biotechnol. Biochem.* **70**, 3073–3076 (2006). 17151450 10.1271/bbb.60407

[33] Brusselaers, N., Shrestha, S., van de Wijgert, J. & Verstraelen, H. Vaginal dysbiosis and the risk of human papillomavirus and cervical cancer: systematic review and meta-analysis. *Am. J. Obstet. Gynecol.* **221**, 9–18.e8 (2019). 30550767 10.1016/j.ajog.2018.12.011

[34] Santella, B. et al. Microbiota and HPV: The role of viral infection on vaginal microbiota. *J. Med. Virol.* **94**, 4478–4484 (2022). 35527233 10.1002/jmv.27837 PMC9544303

[35] Mitra, A. et al. The vaginal microbiota and innate immunity after local excisional treatment for cervical intraepithelial neoplasia. *Genome Med.* **13**, 176 (2021). 34736529 10.1186/s13073-021-00977-w PMC8567681

[36] Preisler, S. et al. Prevalence of human papillomavirus in 5,072 consecutive cervical SurePath samples evaluated with the Roche cobas HPV real-time PCR assay. *PLoS ONE* **8**, e59765 (2013). 23533648 10.1371/journal.pone.0059765 PMC3606112

[37] Moro-Rodriguez, E. & Alvarez-Fernandez, E. Losses of expression of the antigens A, Lea and Lex and over-expression of Ley in carcinomas and HG-SIL of the uterine cervix. *Diagn. Pathol.* **3**, 38 (2008). 18786253 10.1186/1746-1596-3-38 PMC2551588

[38] Stubbe Teglbjaerg, C., Norrild, B. & Dabelsteen, E. Changes of blood group antigens in premalignant and malignant lesions of the human exocervix. *Acta Pathol. Microbiol. Immunol. Scand. A* **93**, 149–151 (1985). 4013740 10.1111/j.1699-0463.1985.tb03933.x

[39] Parish, W. E., Carron-Brown, J. A. & Richards, C. B. The detection of antibodies to spermatozoa and to blood group antigens in cervical mucus. *J. Reprod. Fertil.* **13**, 469–483 (1967). 4165909 10.1530/jrf.0.0130469

[40] Branch, D. R. Anti-A and anti-B: what are they and where do they come from? *Transfusion* **55**, S74–S79 (2015). 26174901 10.1111/trf.13087

[41] Mironov, A. A., Savin, M. A., Zaitseva, A. V., Dimov, I. D. & Sesorova, I. S. Mechanisms of formation of antibodies against blood group antigens that do not exist in the body. *Int J. Mol. Sci.* **24**, 15044 (2023). 37894724 10.3390/ijms242015044 PMC10606600

[42] Bunker, J. J. et al. Natural polyreactive IgA antibodies coat the intestinal microbiota. *Science* **358**, eaan6619 (2017). 28971969 10.1126/science.aan6619 PMC5790183

[43] Puga, I. & Cerutti, A. Protection by natural IgG: a sweet partnership with soluble lectins does the trick! *EMBO J.* **32**, 2897–2899 (2013). 24162726 10.1038/emboj.2013.235 PMC3831308

[44] Arnolds, K. L., Martin, C. G. & Lozupone, C. A. Blood type and the microbiome- untangling a complex relationship with lessons from pathogens. *Curr. Opin. Microbiol.* **56**, 59–66 (2020). 32663769 10.1016/j.mib.2020.06.008 PMC10104170

[45] Lee, S. et al. Glycan-mediated molecular interactions in bacterial pathogenesis. *Trends Microbiol.* **30**, 254–267 (2022). 34274195 10.1016/j.tim.2021.06.011 PMC8758796

[46] MacIntyre, D. A. et al. The vaginal microbiome during pregnancy and the postpartum period in a European population. *Sci. Rep.* **5**, 8988 (2015). 25758319 10.1038/srep08988 PMC4355684

[47] Chakravorty, S., Helb, D., Burday, M., Connell, N. & Alland, D. A detailed analysis of 16S ribosomal RNA gene segments for the diagnosis of pathogenic bacteria. *J. Microbiol. Methods* **69**, 330–339 (2007). 17391789 10.1016/j.mimet.2007.02.005 PMC2562909

[48] Bolyen, E. et al. Reproducible, interactive, scalable and extensible microbiome data science using QIIME 2. *Nat. Biotechnol.* **37**, 852–857 (2019). 31341288 10.1038/s41587-019-0209-9 PMC7015180

[49] Fettweis, J. M. et al. Species-level classification of the vaginal microbiome. *BMC Genomics* **13**, S17 (2012). 23282177 10.1186/1471-2164-13-S8-S17 PMC3535711

[50] France, M. T. et al. VALENCIA: a nearest centroid classification method for vaginal microbial communities based on composition. *Microbiome* **8**, 166 (2020). 33228810 10.1186/s40168-020-00934-6 PMC7684964

[51] North, S. J. et al. Mass spectrometric analysis of mutant mice. *Methods Enzymol.* **478**, 27–77 (2010). 20816474 10.1016/S0076-6879(10)78002-2

[52] Song, X. et al. Novel fluorescent glycan microarray strategy reveals ligands for galectins. *Chem. Biol.* **16**, 36–47 (2009). 19171304 10.1016/j.chembiol.2008.11.004 PMC2662446

[53] Chai, W., Stoll, M., Galustian, C., Lawson, A. & Feizi, T. Neoglycolipid technology: deciphering information content of glycome. *Methods Enzymol.* **362**, 160–195 (2003). 12968363 10.1016/S0076-6879(03)01012-7

[54] Blixt, O. et al. Printed covalent glycan array for ligand profiling of diverse glycan binding proteins. *Proc. Natl Acad. Sci. USA* **101**, 17033–17038 (2004). 15563589 10.1073/pnas.0407902101 PMC534418

[55] Wu, N. et al. Glycan markers of human stem cells assigned with beam search arrays. *Mol. Cell. Proteom. : MCP* **18**, 1981–2002 (2019). 31308253 10.1074/mcp.RA119.001309 PMC6773554

[56] Liu, Y. et al. The minimum information required for a glycomics experiment (MIRAGE) project: improving the standards for reporting glycan microarray-based data. *Glycobiology* **27**, 280–284 (2017). 27993942 10.1093/glycob/cww118 PMC5444268

[57] Liu, Y. et al. Neoglycolipid-based oligosaccharide microarray system: preparation of NGLs and their noncovalent immobilization on nitrocellulose-coated glass slides for microarray analyses. *Methods Mol. Biol. (Clifton, NJ)* **808**, 117–136 (2012). 10.1007/978-1-61779-373-8_8 22057521

[58] Akune, Y. et al. CarbArrayART: a new software tool for carbohydrate microarray data storage, processing, presentation, and reporting. *Glycobiology* **32**, 552–555 (2022). 35352122 10.1093/glycob/cwac018 PMC9191619

[59] R Development Core Team. *R: A Language and Environment for Statistical Computing* (R Foundation for Statistical Computing, 2011) http://www.R-project.org.

