# Supplementary material for: ABO blood group antigens influence host–microbe interactions and risk of early spontaneous preterm birth
Source: NPJ Biofilms Microbiomes. 2025 Sep 10;11:170. doi: 10.1038/s41522-025-00783-x (PMC12423329; doi:10.1038/s41522-025-00783-x)
Supplement: Supplementary file 1 — Supplementary information [file 41522_2025_783_MOESM1_ESM.pdf]

Supplementary Information

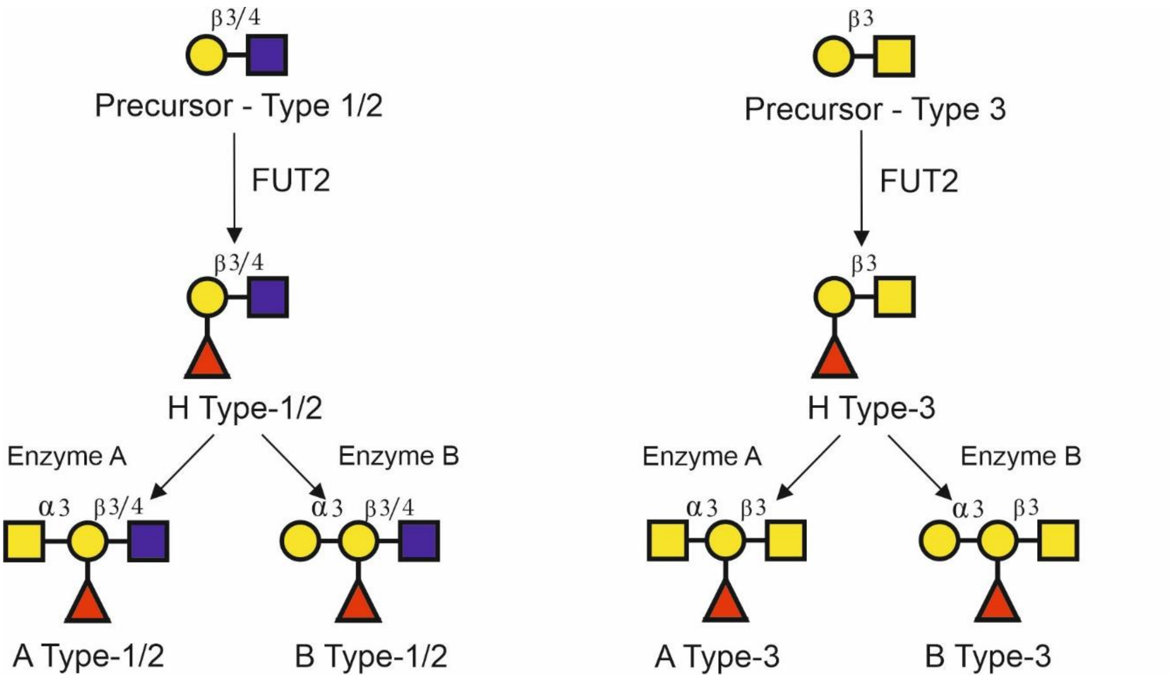

Supplementary Figure 1: Synthesis of ABO blood group antigens

Schematic representation of the formation of ABH blood group antigens on N and O-glycan structures from Type 1, Type 2 and Type 3 chain precursors by glycosyltransferases (FUT2, A and B enzymes).

Supplementary Table 1 Demographic Data – General Obstetric Population (iCARE Cohort)

		A	AB	B	O	Total	p
**Total N (%)**		24911 (33.3)	4301 (5.7)	13963 (18.6)	31738 (42.4)	74913	
**Gravidity**	Median (IQR)	2.0 (1.0 to 3.0)	2.0 (1.0 to 3.0)	2.0 (1.0 to 3.0)	2.0 (1.0 to 3.0)	2.0 (1.0 to 3.0)	<0.001
**Parity**	Median (IQR)	0.0 (0.0 to 1.0)	0.0 (0.0 to 1.0)	0.0 (0.0 to 1.0)	0.0 (0.0 to 1.0)	0.0 (0.0 to 1.0)	<0.001
**Age**	Median (IQR)	33.0 (30.0 to 37.0)	33.0 (30.0 to 37.0)	33.0 (29.0 to 37.0)	33.0 (30.0 to 37.0)	33.0 (30.0 to 37.0)	0.020
**BMI**	Median (IQR)	24.1 (21.5 to 27.7)	24.2 (21.5 to 27.8)	24.4 (21.7 to 28.1)	24.4 (21.7 to 28.1)	24.3 (21.6 to 27.9)	<0.001
**Smoking status**	Yes	928 (3.9)	144 (3.5)	394 (2.9)	1211 (4.0)	2677 (3.7)	<0.001
	No	22721 (95.6)	3978 (96.2)	12928 (96.6)	29037 (95.6)	68664 (95.8)	
	Unknown	119 (0.5)	15 (0.4)	57 (0.4)	122 (0.4)	313 (0.4)	
**Rh status**	Negative	2821 (11.3)	413 (9.6)	1277 (9.2)	3177 (10.0)	7688 (10.3)	<0.001
	Positive	22066 (88.7)	3880 (90.4)	12672 (90.8)	28528 (90.0)	67146 (89.7)	
**Ethnicity**	White	10627 (42.7)	1372 (31.9)	3468 (24.9)	11549 (36.4)	27016 (36.1)	<0.001
	Asian	3239 (13.0)	1039 (24.2)	3953 (28.3)	4564 (14.4)	12795 (17.1)	
	Black	2200 (8.8)	295 (6.9)	1462 (10.5)	4622 (14.6)	8579 (11.5)	
	Other	3298 (13.2)	618 (14.4)	1928 (13.8)	4093 (12.9)	9937 (13.3)	
	Mixed	875 (3.5)	157 (3.7)	465 (3.3)	1205 (3.8)	2702 (3.6)	
	Not Stated	4656 (18.7)	819 (19.0)	2679 (19.2)	5690 (17.9)	13844 (18.5)	
**Previous Cervical Treatment**	No	24015 (96.4)	4187 (97.3)	13611 (97.5)	30762 (96.9)	72575 (96.9)	<0.001
	Yes	896 (3.6)	114 (2.7)	352 (2.5)	976 (3.1)	2338 (3.1)	
**Previous sPTB**	No	24517 (98.4)	4228 (98.3)	13758 (98.5)	31206 (98.3)	73709 (98.4)	0.394
	Yes	394 (1.6)	73 (1.7)	205 (1.5)	532 (1.7)	1204 (1.6)	
**Previous MTL**	No	24659 (99.0)	4258 (99.0)	13796 (98.8)	31426 (99.0)	74139 (99.0)	0.207

Supplementary Table 2 Demographic Data High-Risk Population (VMET2 Cohort)

*Blood group AB not included in further analysis due to small n.

Demographics High-Risk		A	AB*	B	O	Total	p
Total N (%)		721 (34.6)	101 (4.8)	368 (17.7)	894 (42.9)	2084	
Gravida	Median (IQR)	2.0 (2.0 to 4.0)	3.0 (2.0 to 3.0)	3.0 (2.0 to 4.0)	3.0 (2.0 to 4.0)	2.0 (2.0 to 4.0)	0.439
Parity	Median (IQR)	1.0 (0.0 to 1.0)	0.0 (0.0 to 1.0)	1.0 (0.0 to 1.0)	1.0 (0.0 to 1.0)	1.0 (0.0 to 1.0)	0.279
Age	Median (IQR)	34.0 (31.0 to 37.0)	34.0 (31.0 to 37.2)	34.0 (31.0 to 37.0)	34.0 (31.0 to 37.0)	34.0 (31.0 to 37.0)	0.390
BMI	Median (IQR)	24.0 (21.4 to 27.4)	24.0 (21.0 to 27.2)	24.0 (21.0 to 28.0)	24.0 (21.1 to 28.0)	24.0 (21.2 to 28.0)	0.807
Smoking Status	Yes	19 (2.9)	2 (2.2)	4 (1.2)	23 (2.8)	48 (2.5)	0.375
	No	642 (97.1)	88 (97.8)	332 (98.8)	788 (97.2)	1850 (97.5)	
Rh Status	Positive	592 (87.7)	80 (84.2)	320 (92.0)	741 (89.3)	1733 (89.0)	0.089
	Negative	83 (12.3)	15 (15.8)	28 (8.0)	89 (10.7)	215 (11.0)	
Ethnicity	White	488 (68.4)	67 (66.3)	151 (41.9)	519 (58.4)	1225 (59.4)	<0.001
	Asian	75 (10.5)	14 (13.9)	96 (26.7)	127 (14.3)	312 (15.1)	
	Black	84 (11.8)	9 (8.9)	77 (21.4)	169 (19.0)	339 (16.4)	
	Other	66 (9.3)	11 (10.9)	36 (10.0)	74 (8.3)	187 (9.1)	
Risk Factor for sPTB	Cervical Treatment	317 (44.0)	41 (40.6)	103 (28.0)	331 (37.0)	792 (38.0)	0.001
	Previous PTB/MTL	329 (45.6)	55 (54.5)	224 (60.9)	471 (52.7)	1079 (51.8)	
	Incidental Short Cervix	25 (3.5)	3 (3.0)	15 (4.1)	36 (4.0)	79 (3.8)	
	Uterine Anomaly	18 (2.5)	0 (0.0)	9 (2.4)	15 (1.7)	42 (2.0)	
	Other	32 (4.4)	2 (2.0)	17 (4.6)	41 (4.6)	92 (4.4)	

Supplementary Table 3: Comparison of cervical cerclage rates according to ABO blood group for general population

Bg1	Bg2	n	OR	95% CI-low	95% CI-high	p
**A**	**AB**	28235	1.074637	0.7420190	1.520695	0.654
**A**	**B**	37540	1.569547	1.2793821	1.923957	<0.0001
**A**	**O**	54715	1.160950	0.9707688	1.390565	0.101

Supplementary Table 4: Comparison of pregnancy outcomes between ABO blood groups for high-risk population

Outcome	Bg1	Bg2	n	OR	95% CI-low	95% CI-high	p
Short Cervix	**A**	**AB**	788	1.0986431	0.6270774	1.862975	0.3590
Short Cervix	A	B	1033	1.3590040	0.9949446	1.851223	0.0219*
Short Cervix	**A**	**O**	1547	1.1966620	0.9372897	1.530107	*0.0692*
MTL	**A**	**AB**	788	0.5226129	0.0122252	3.511800	0.7370
MTL	**A**	**B**	1033	1.6281316	0.6610056	3.908602	0.1140
MTL	**A**	**O**	1547	1.1660654	0.5553809	2.515488	0.0331*
28 weeks	**A**	**AB**	788	1.0069843	0.1892471	3.452260	0.4960
28 weeks	A	B	1033	2.0157211	1.0376553	3.905410	0.0111*
28 weeks	**A**	**O**	1547	1.3847387	0.7869069	2.489996	0.1170
34 weeks	**A**	**AB**	788	0.9386474	0.3186318	2.289103	0.5560
34 weeks	**A**	**B**	1033	1.6347652	1.0060268	2.638194	0.0163*
34 weeks	**A**	**O**	1547	1.4047456	0.9513009	2.093238	0.0372*
37 weeks	**A**	**AB**	788	0.8812251	0.4452788	1.634576	0.6600
37 weeks	**A**	**B**	1033	1.1715361	0.8211848	1.661428	*0.0790*
37 weeks	**A**	**O**	1547	0.9549956	0.7215964	1.265443	*0.0870*

Supplementary Table 5: Bacterial species and strains used for glycan binding arrays

Species	Strain ID	Abbreviation	Source	Culturing media solid	Culturing media liquid
Lactobacillus crispatus	UMB1398	*L. crispatus 1398*	Wolfe Laboratory*	MRS	MRS
Lactobacillus iners	DSM13335	*L. iners 13335*	DSMZ	Columbia blood agar	VMM
Gardnerella vaginalis	UMB0540	*G. vaginalis 540*	Wolfe Laboratory*	Columbia blood agar	VMM
Streptococcus agalactiae	UMB0776	*S. agalactiae 776*	Wolfe Laboratory*	BHI	BHI

*Kindly provided by Alan Wolfe and Trevor Lawley. *Nat. Commun.* **9,** 1557 (2018).

Supplementary Table 6: Glycan library of sequency defined ABH probes

Supplementary table 2: List of glycan probes used

Glycan Probe information			Subarray information***			
Probe Id	Probe Name*	Probe Structure**	Subarray	Position#	Subarray	Position#
3158	A-T1-Hexa-AEAB	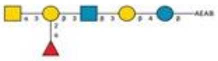	1	9	2	26
3159	A-T2-Hexa-AEAB	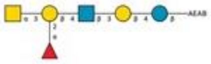	1	12	2	27
3161	B-T1-Hexa-AEAB	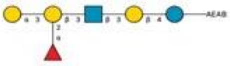	1	15	2	29
3162	B-T2-Hexa-AEAB	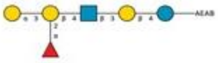	1	18	2	30
3330	LNFPI-AEAB	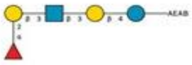	1	21	2	58
3164	LNnFPI-AEAB	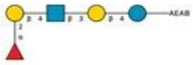	1	24	2	32

FOOTNOTE:
*The probe names include an abbreviated description of the glycan moiety followed by the chemical linker abbreviation: AEAB: N-(aminoethyl)-2-aminobenzamide
**The probe structure is shown using the following monosacharide legend:
Galactose, Glucose, N-Acetyl glucosamine, Fucose
***Refers to the subarray/subarrays in which the probe is included. See MIRAGE document (Suppl. File 1).
# Refers to position on subarray. See MIRAGE document (Suppl. File 1).

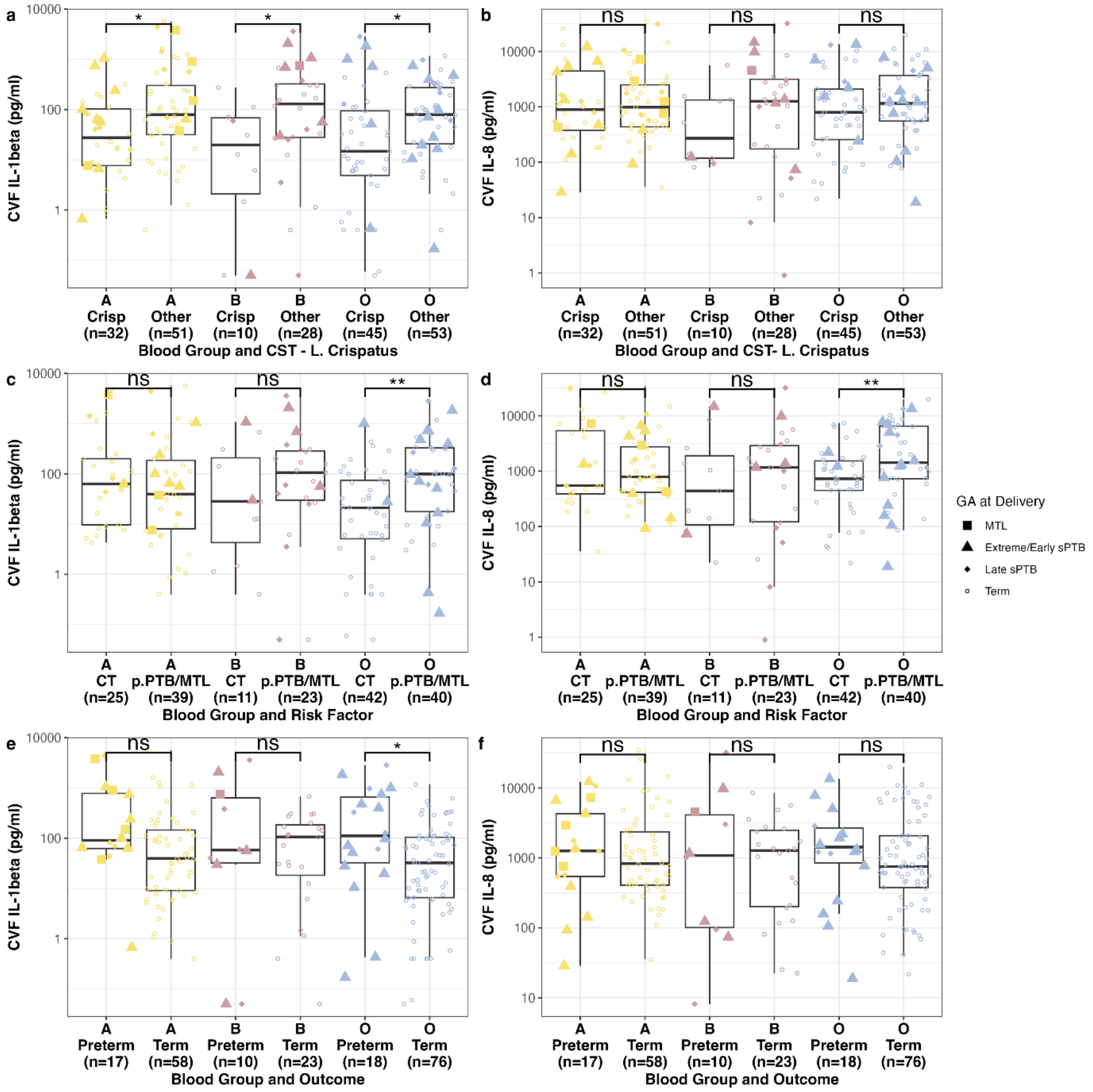

Supplementary Figure 2: Cervicovaginal Fluid (CVF) cytokine concentrations by ABO blood group phenotype at timepoint 1 ($12+^{0}$ – $15+^{6}$)

a) CVF IL-1β (pg/ml) and community state type (CST) (n=219). **b)** CVF IL-8 (pg/ml) and CST (n=219). **c)** CVF IL-8 (pg/ml) and risk factor (n=180). **d)** CVF IL-8 (pg/ml) and risk factor (n=180). **e)** CVF IL-1β (pg/ml) and sPTB (iPTB excluded) (n=202). **f)** CVF IL-8(pg/ml) and sPTB (iPTB excluded) (n=202). Wilcoxon Signed-Rank Test adjusted for multiple comparisons ($p<0.05$ - *, $p<0.01$ - **, $p<0.001$ - ***, $p<0.0001$ - ****).

Supplementary File 1. Supplementary glycan microarray document based on MIRAGE Glycan Microarray guidelines (doi:10.3762/mirage.3).

Classification	Guidelines
**1. Sample: Glycan Binding Sample**	
Description of Sample	Sample name Bacteria:<br>*L. crispatus* 1398<br>*L. iners* 13335 (DSMZ)<br>*G. vaginalis* 540<br>*S. agalactiae* 776<br>Origin:<br>The bacterial isolates used in this study were commercially sourced (DSMZ) or patient-derived strains provided by the Lawley and Wolfe lab (Thomas-White *et al.* Nature communications 2018).<br>Method of preparation: Please see ***Bacterial culture and labelling*** *section* in Online Methods.
Sample modifications	Fluorescent staining of live bacterial cultures with a fluorescent compound is as described in ***Bacterial culture and labelling*** section in Online Methods.
Assay protocol	Microarray analyses of lectins and anti-carbohydrate specific antibodies were performed essentially as described (Liu *et al.*, Methods Mol. Biol. 2012), modifications of the protocol are: no blocking was performed for analysis using covalent microarrays.<br><br>Microarray analyses of fluorescently labelled bacteria was performed as follows. Fluorescently labelled bacterial cultures at OD=1 were resuspended in binding buffer (10 mM Hepes, 150 mM NaCl, and 5 mM $CaCl_2$, pH 7 or supplemented with HCl to reach pH 4) and were used for overlays on glycan microarrays. 100 µL of bacterial suspension was applied to the incubation chamber with the microarrays, incubated for 1 h at room temperature under mild agitation on an oscillating platform and washed three times with binding buffer followed by two washes with HPLC grade water to remove salts from the array. Slides were dried under a mild nitrogen flow and scanned for quantitation as described below.
**2. Glycan Library**	
Glycan description for defined glycans	Six blood group related glycan probes were used in glycan microarray analyses. The probe names and sequences are in **Supplementary Data 1**. Details about glycan origin and preparation is in Online Methods. Two

	subarrays, printed at different times with the same set of blood group related probes were used for this work, please see **Supplementary Data 1**.
Glycan description for undefined glycans	N/A
Glycan modifications	The preparation and characterization of the amino-terminating sequence-defined oligosaccharide probes are detailed in Online Materials and Methods.
**3. Printing Surface; e.g., Microarray Slide**	
Description of surface	NHS-ester functionalized hydro-polymer
Manufacturer	Nexterion™ 3-D Hydrogel (H) glass microarray slide from the SCHOTT Group (Jena, Germany)
Custom preparation of surface	N/A
Covalent Immobilisation	The sequence-defined amino-terminating oligosaccharide probes were printed at concentrations of 300 µM in phosphate buffer (0.1M Na_2HPO4/NaH_2PO4 buffer, pH 8.0).<br>Upon completion of the printing process, the slides were immersed for 1 hour in a 100 mM Sodium Borate buffer at pH 8.5 containing 50 mM Ethanolamine. Subsequently, the slides are washed four times with a PBS and Tween 20% solution, followed by a wash with Nanopure water. The slides were then dried and stored at 20°C.
**4. Arrayer (Printer)**	
Description of Arrayer	Nano-Plotter 2.1 (GeSim, Radeberg, Germany)
Dispensing mechanism	Non-contact liquid delivery with four dispensing tips.
Glycan deposition	Each glycan probe was printed in quadruplicate, with approximately 0.7 nl per spot (two droplets of ~ 0.33 nl each).
Printing conditions	Glycan probes at indicated concentration in phosphate buffer (pH 8.0) were transferred to a 384-well microtiter plate for printing. The printing processes were conducted under ambient conditions with relative humidity maintained between 55% and 60%, and completed within 8 hours.<br>The positional marker was prepared by diluting Streptavidin-Alexa Fluor 647 conjugate in the same phosphate buffer to a concentration of 0.1 µg/µl.

**5. Glycan Microarray with “Map”**	
Array layout	Two subarrays were used, subarray 1 and subarray 2. Subarrays 1 and 2 were printed at different times with the same set of blood group related probes. The composite glycan probes, structures, their positions on the subarrays and the quality control (QC) data can be found in **Supplementary Data 1.**<br>Slide format: Each array slide contained 16 identical subarrays (pads). Each pad was set up for printing 69 probes maximum, each in quadruplicate (four spots for one probe in a row); up to 276 spots (16 Columns x17 Rows) in total in each pad.
Glycan identification and quality control	The quality control of the glycan microarrays was carried out with the proteins listed below. Details on the origin of these and concentrations used for analysis along with the binding results can be found in **Supplementary Data 1:**<br>(i) a panel of biotinylated plant lectins (Vector Laboratories), e.g. *Ricinus Communis* Agglutinin I (RCA_{120}), *Aleuria aurantia* lectin (AAL), Concanavalin A (ConA), wheat germ agglutinin (WGA) and Ulex Europaeus Agglutinin (UEA).<br>(ii) anti-carbohydrate antibodies, Anti- Blood group A (clone Z2A), Anti- Blood group A (cloneT36), Anti- Blood group B (HEB29), Anti- Blood group B (BG3), Anti- Blood group H type 1 (clone 17-206), Anti- Blood group H type 2 (BE2).

**Anti-carbohydrate antibodies and plant lectins:**				
Antibody	*Clone*	*Other designation*	*Vendor or collaborator*	*Isotype*
Anti- Blood group A	Z2A	-	Sigma	Mouse IgM
Anti- Blood group A	T36	BG2	Biolegend	Mouse IgG
Anti- Blood group B	-	HEB29	Abcam	Mouse IgM
Anti- Blood group B	-	BG3	Biolegend	Mouse IgM
Anti- Blood group H type 1	17-206	-	Invitrogen	Mouse IgG
Anti- Blood group H type 2	-	BE2*	Henrik Clausen	Mouse IgM
Plant Lectin		*Other designation*	*Vendor*	*Used conc.*
Aleuria Aurantia Lectin		AAL	Vector Laboratories	20 μg/mL

	Concanavalin A	ConA	Vector Laboratories	5 µg/mL
	Ricinus Communis Agglutinin I	RCA 120	Vector Laboratories	5 µg/mL
	Wheat Germ Agglutinin	WGA	Vector Laboratories	20 µg/mL
	Ulex Europaeus Agglutinin I	UEA-I	Vector Laboratories	50 µg/mL

**6. Detector and Data Processing**	
Scanning hardware	GenePix 4300A (Molecular Devices)
Scanner settings	Scanning resolution: 5 µm / pixel<br>Laser channel: Red (scan wavelength 635 nm)<br>PMT: 400<br>Scan power: 100% was used to record the optimal scan images without spot saturation.
Image analysis software	GenePix® Pro 7 (Molecular Devices)
Data processing	The gpr files were entered into CArbArrayART (Akune *et al*. Glycobiology 2022). The background substrated mean of the quadruplicates on array was used for all analysis presented in this work. SD of quadruplicates is provided in **Supplementary Data 1.** % MAX, RANK and Average RANK was calculated as indicated in **Supplementary Data 1** and summarised in **Figure 3e.**
**7. Glycan Microarray Data Presentation**	
Data presentation	The microarray binding results are presented as:<br>(i) Heatmaps with Average RANK scores of relatively binding intensities whose calculations can be found in **Supplementary Data 1.**
**8. Interpretation and Conclusion from Microarray Data**	

Data interpretation	No software or algorithms were used to interpret processed data.
Conclusions	*All strains tested, ie: L. crispatus* 1398, *L. iners* 13335 (DSMZ), *G. vaginalis* 540 and *S. agalactiae* 776, bind to Blood group A, B and H on type 2 backbones glycan probes and Blood group B and H on type 1 backbones with varying strengths. Poor or no binding to Blood group A on a type 1 backbone was observed by the bacteria strains tested. Additionally, differential binding is observed at different experimental pH by all strains tested.

Supplementary Data 1
RANK calculation of ABO See Separate Supplementary Data File 1
